# Screening Autophagy‐Related DKD Biomarkers Based on Bulk RNA and Single‐Cell Analysis and Uncovering Their Regulatory Mechanisms in DKD Renal Fibrosis

**DOI:** 10.1155/jdr/3768039

**Published:** 2026-04-07

**Authors:** Qin Wang, Xiaoqi Li, Wen Ye, Qi Wang, Xianjin Bi

**Affiliations:** ^1^ Department of Geriatric Integrative, The Second Affiliated Hospital of Xinjiang Medical University, Urumqi, Xinjiang, China, xjmu.edu.cn; ^2^ Department of Geriatric Medicine, The Second Affiliated Hospital of Xinjiang Medical University (The Second Clinical Medical College), Xinjiang Medical University, Urumqi, Xinjiang, China, xjmu.edu.cn; ^3^ Department of Nephrology, The Second Affiliated Hospital of Xinjiang Medical University, Urumqi, Xinjiang, China, xjmu.edu.cn; ^4^ Department of Preventive Medicine; Xinqiao Hospital, Army Medical University, Chongqing, China, tmmu.edu.cn; ^5^ Department of Nephrology, the Key Laboratory for the Prevention and Treatment of Chronic Kidney Disease of Chongqing, Kidney Center of PLA, Xinqiao Hospital, Army Medical University, Chongqing, China, tmmu.edu.cn; ^6^ Department of Cardiology, People’s Liberation Army Joint Logistic Support Force 945th Hospital, Ya’an, China

**Keywords:** autophagy, bulk RNA analysis, diabetic kidney disease (DKD), renal fibrosis, single-cell analysis

## Abstract

**Background:**

Diabetic kidney disease (DKD) is a common diabetes complication that increases global morbidity and mortality. To identify DKD biomarkers and explore autophagy‐related mechanisms to find potential therapeutic targets for DKD treatment.

**Methods:**

After standardizing and analyzing the GSE142025 and Merged_GSE47183_GSE32591 datasets, DKD‐related transcriptional changes (DEGs) were identified using computational methods, volcano plots, and heatmaps. Functional enrichment analysis of GO and KEGG pathways explored the role of autophagy‐related genes. Key genes were identified through a PPI network, and potential DKD biomarkers were selected using machine learning. ROC curve analysis evaluated the biomarkers’ diagnostic effectiveness. qPCR and immunohistochemical staining confirmed biomarker expression in DKD mouse kidneys. snRNA‐seq identified cell‐specific transcriptional profiles and signaling networks. Pseudotemporal analysis highlighted DKD‐related PCT cell dynamics.

**Results:**

This study showed autophagy‐related gene enrichment in pathways like inflammatory response and TNF/NF‐κB signaling. Four biomarkers (COL1A2, CSF1R, PTPRC, and TYROBP) showed diagnostic potential for DKD. Immune cell infiltration analysis revealed differences between DKD and control groups. qPCR and staining indicated significant upregulation of these biomarkers. snRNA‐seq identified cell clusters and DEGs, with altered signaling pathways and PCT cell communication. Network centrality analysis highlighted the differing roles of PCT in cell communication between control and DKD groups. Pseudotime trajectory analysis and BEAM revealed potential regulatory mechanisms in PCT cell differentiation and development in DKD.

**Conclusions:**

This study offers new insights into DKD biomarkers and autophagy‐related mechanisms, suggesting potential therapeutic targets and laying the groundwork for future research and treatment strategies.

## 1. Introduction

Diabetic kidney disease (DKD) represents the leading diabetes‐induced microangiopathy, with its global incidence escalating in parallel with the expanding diabetic population [1]. Studies indicate that ~30% to 40% of the diabetic cohort progress to DKD manifestations, which not only significantly elevates morbidity and mortality among patients but also imposes a substantial burden on public health [2]. The pathological mechanisms of DKD are complex and involve multiple factors, including hyperglycemia, inflammatory responses, and oxidative stress [3]. The clinical manifestations of DKD typically progress through several stages. In the early stages, patients may exhibit microalbuminuria, often undetectable in routine urine tests. As the disease advances, patients develop overt proteinuria and may even progress to renal failure [4]. Clinically, DKD patients often present with symptoms such as hypertension, edema, and fatigue, which intensify as renal function declines [5]. Approximately 40% of individuals with diabetes will end up with end‐stage renal disease, which will require either dialysis or kidney transplant [6]. Furthermore, clinical studies have shown that patients with DKD are also at increased risk for cardiovascular diseases, which is closely associated with renal injury and systemic inflammatory responses [7]. Therefore, early identification and intervention in DKD are pivotal for optimizing disease trajectories.

In recent years, autophagy, as a crucial intracellular degradation mechanism, has increasingly garnered attention from researchers. Autophagy primarily involves encapsulating damaged or excessive CCs within autophagosomes, which subsequently fuse with lysosomes for degradation [8]. This mechanism serves as a central regulator of cellular metabolic equilibrium, orchestrates stress adaptation under nutrient scarcity, and mediates the elimination of dysfunctional cytoplasmic components [9]. Autophagy is pivotal in sustaining cellular function and internal environmental stability, with its dysregulation being mechanistically linked to the pathogenesis of multiple disorders [10–12]. The dysregulation of autophagy has been mechanistically implicated in driving the pathogenesis of DKD [13]. Autophagy not only mediates the removal of compromised cytoplasmic components but also serves as a central regulator of cellular metabolic equilibrium, counteracting redox imbalance while coordinating immunomodulatory pathways [14]. Research has demonstrated that under diabetic conditions, autophagic flux is impaired within renal tubular epithelial cells and podocytes, resulting in functional impairment and renal injury [15, 16]. Defects in autophagy may result in the accumulation of intracellularly damaged substances, accelerating renal pathology characterized by tubular atrophy and interstitial fibrosis [17]. Therefore, exploring the role of autophagy in DKD and its potential therapeutic targets may uncover novel strategies and approaches toward mitigating and managing DKD.

In our study, DKD‐related differential expression genes (DEGs) were screened from datasets GSE142025 and Merged_GSE47183_GSE32591, following normalization and PCA. Volcano plots and heatmaps identified significant DEGs, with GO and KEGG enrichment analyses revealing enrichment in inflammatory response, leukocyte migration, and TNF/NF‐kappa B signaling among autophagy‐related genes. PPI network analysis screened out hub genes, leading to the selection of four potential DKD biomarkers (COL1A2, CSF1R, PTPRC, and TYROBP) via machine learning algorithms. ROC curves validated their expression and diagnostic performance. Further KEGG and Gene Set Enrichment Analysis (GSEA) analyses provided insights into biomarker‐associated pathways. In DKD mice, qPCR and immunohistochemical staining demonstrated a notable upregulation of the expression of four biomarker genes in their kidneys. Immune cell infiltration analysis using CIBERSORT identified differences between DKD and control groups, while snRNA‐seq annotated cell clusters and DEGs. Cell–cell communication analysis showed alterations in signaling pathways and PCT cell communication patterns. Network centrality analysis highlighted differences in PCT’s role in cell communication between control and DKD groups. Collectively, the present work elucidates critical perspectives on DKD biomarkers and pathophysiological pathways involving autophagy, establishing a foundation for subsequent investigations and therapeutic innovation.

## 2. Methods

### 2.1. Data Collection and Preprocessing

Gene expression profiling data related to DKD was sourced from the NCBI Gene Expression Omnibus (GEO) database (https://www.ncbi.nlm.nih.gov/geo/). The GEOquery package [18] was employed to download the following datasets from the GEO database(https://www.ncbi.nlm.nih.gov/U): GSE142025, GSE47183, GSE32591, and GSE104948. Among them, GSE142025 is an RNA‐seq dataset, while GSE47183, GSE32591, and GSE104948 are microarray datasets. Specifically, the GSE142025 dataset contains renal biopsy samples from 27 DKD patients and 9 control samples. The GSE47183 dataset comprises glomerular biopsy samples from 14 DKD patients. The GSE32591 dataset includes glomerular biopsy tissue samples from 14 healthy individuals. SE104948 dataset is used as an external validation set. Next, for the RNA‐seq dataset GSE142025, we used the DESeq2 R package [19] to perform normalized counts and differential expression analysis. For the microarray datasets GSE47183, GSE32591, and GSE104948, we used the normalizeBetweenArrays function from the limma package [20] to standardize the datasets. The datasets GSE47183 and GSE32591 were merged into “Merged_GSE47183_GSE32591” and batch effects were removed, followed by another round of standardization. The standardized datasets underwent principal component analysis (PCA), and the ggplot2 package was used to create boxplots and PCA plots for visualizing sample distribution and clustering within the datasets.

### 2.2. Identification of DEGs

The process of identifying DEGs started with a differential analysis of the GSE142025 and Merged_GSE47183_GSE32591 datasets utilizing the DESeq2 R package and limma package [21], respectively. The Benjamini–Hochberg multiple testing correction was applied to calculate the false discovery rate (FDR), and genes with FDR < 0.05 were considered significantly differentially expressed. By applying the criteria of |log2FC|>1 and Padj.<0.05, we discovered statistically significant DEGs in both datasets. Subsequently, we used the ggplot2 package to generate volcano plots and the ComplexHeatmap package to create heatmaps [22] to illustrate the expression levels of the top 20 genes that are significantly upregulated and downregulated across both datasets.

### 2.3. Acquisition of Autophagy‐Related Genes

We explored various databases to further investigate the molecular mechanisms of autophagy in DKD, sourcing autophagy‐related genes from the PubMedGene database (https://www.ncbi.nlm.nih.gov/gene/), Genecard database (https://www.genecards.org/), GSEA database (https://www.gsea-msigdb.org/), and autophagy‐related websites (http://www.autophagy.lu/, http://hamdb.scbdd.com/home/index/).

### 2.4. GSEA

To understand the systemic biological disorders of DKD, we performed GSEA, and we employed the clusterProfiler package [23] to perform GSEA [24]. Specific parameters were determined in this analysis to screen for pathways with notable enrichment, as follows: species: *Homo sapiens* (human); reference gene set source R package: msigdbr; ID conversion R package: org.Hs.eg.db. The criteria for filtering the enrichment analysis results included FDR < 0.25, and *p*.adjust value < 0.05.

### 2.5. GO and KEGG Enrichment Analysis

DEGs were identified common in DKD by intersecting them with genes related to autophagy. Visualization was done using Venn diagrams created with the ggplot2 and VennDiagram packages [version 1.7.3]. Afterwards, GO functional enrichment analysis was conducted on *Homo sapiens* background genes. Detailed annotations and classifications were offered according to the three major functional categories of genes: biological process (BP), cellular component (CC), and molecular function (MF). Additionally, to discover key biological pathways potentially involved by the DEGs, we conducted KEGG pathway enrichment analysis [25–27]. The org.Hs.eg.db package was used to convert the input gene lists to appropriate IDs. Enrichment analysis was then performed using the clusterProfiler package [version 4.4.4] [23]. The GOplot package [version 1.0.2] was utilized to calculate the z‐score for each enrichment term, assessing the significance of the enrichment results [28]. A threshold of *p*  < 0.05 and FDR <0.2 was adopted to filter out statistically significant and biologically meaningful results [29]. The FDR threshold was set to <0.2, a threshold that is more lenient than the conventional < 0.05, in line with common practices in exploratory bioinformatics analyses [30]. This approach aims to reduce the likelihood of Type II errors (false negatives) and to retain a broader set of biologically relevant terms or pathways for hypothesis generation. The subsequent prioritization and interpretation of results were guided by a combination of statistical evidence (both nominal *p*‐value and FDR) and biological context. The complete ranked list is provided in Supporting Information 1: File S1. Finally, the results were visualized using the ggplot2 package.

### 2.6. Protein–Protein Interaction (PPI) Network

Analysis of these intersecting genes was performed using the STRING database (https://string-db.org/) [31]. The threshold for moderate confidence interactions was set at an interaction score greater than 0.4. Interaction node data from STRING was imported into Cytoscape (version 3.10.1) to analyze PPI networks [32]. To further screen for genes that occupy key positions within the network, we applied the MNC, Betweenness, MCC, EPC, and degree algorithms from the CytoHubba plugin to select the top 20 genes in pivotal positions within the PPI network [33]. Subsequently, the results from the five algorithms were intersected to identify hub genes in the PPI network, which were displayed using a Venn diagram. Additionally, we conducted chromosomal localization analysis for the hub genes using the circlize package (version 0.4.16) with the human genome annotation version [*Homo sapiens*] (T2T‐CHM13v2.0) ‐ v2023.6 as the background [34]. Ultimately, we applied the Spearman correlation approach to analyze the connections among these genes and illustrated their degree of association and interaction through a correlation heatmap, using the corrplot package (version 0.92) for visualization.

### 2.7. Machine Learning Identification of Biomarkers for DKD

Potential diagnostic biomarkers were screened independently using LASSO, RF, SVM, and LDA methods. LASSO regression enhances model prediction accuracy through variable selection and coefficient compression by minimizing the coefficients of irrelevant or less critical features to zero while maintaining the valuable ones. Using OLS regression, a regularization term called Lambda is added, which sums up the absolute values of all regression coefficients to manage their size. By making the regularization term coefficient large, some regression coefficients are reduced to zero, aiding in the identification of potential diagnostic biomarkers for DKD. Next, the Random Forest ensemble learning method was used, where features are randomly selected from the feature set, and a large number of decision trees are constructed. An independent sample subset, obtained through bootstrap sampling, is used to train each tree. Individual predictions for new samples are made by each decision tree, with the majority vote determining the final prediction. Following this, the SVM method was utilized to progressively eliminate the least important features through support vector machine recursive feature elimination [35]. Initially, the least critical features are removed based on the SVM model coefficients, and then the remaining features are used to reconstruct the SVM model. This process iteratively reduces the number of included feature genes until the set of features with the lowest model error rate is found. Following this, LDA was used. By estimating the distribution characteristics of each gene in different categories, genes with strong classification ability are calculated and further analyzed as candidate genes. Subsequently, through the feature selection process, genes that contribute most to identifying potential diagnostic biomarkers for DKD are identified. Finally, the results from LASSO, RF, SVM, and LDA were intersected to obtain potential diagnostic biomarkers for DKD. LASSO regression was implemented using the “glmnet” package [36]. Random Forest was implemented using the “randomForest” package [37]. SVM was implemented using the “e1071” package [38] and the “caret” package [39]. LDA was implemented using the “caret” package [39].

### 2.8. Differential Expression Analysis of Potential Diagnostic Biomarkers for DKD

To explore the differential expression of potential diagnostic biomarkers between the experimental and control groups, data were first tested for normality and homogeneity of variance. For data close to a normal distribution (*p*  > 0.05), a *T*‐test was employed to assess differences between groups. When variances were equal (*p*  > 0.05), an independent‐samples *T*‐test was utilized to measure differences in gene expression. Welch’s *T*‐test was applied when the variances between the two groups were statistically different (*p*  < 0.05). The ’ggplot2’ package was used to create violin plots.

### 2.9. Diagnostic ROC Analysis

Diagnostic ROC analysis was conducted separately on the datasets Merged_GSE47183_GSE32591, GSE142025, and GSE104948 using the pROC package. The objective of this analysis was to determine the sensitivity and specificity of the genes mentioned above and to obtain ROC‐related data and information for the predictive variables at their respective cut‐off thresholds, and evaluates how accurately these genes can diagnose DKD. The findings were also displayed using ggplot2. Lastly, GSEA enrichment analysis was performed once more.

### 2.10. Mouse Model

The study involved 8‐week‐old male C57BL/6J mice, which were randomly split into two groups: one receiving streptozocin (STZ) treatment (*n* = 10) and the other receiving citrate buffer treatment (*n* = 10). To simulate the pathophysiological process of human diabetes, they were given intraperitoneal injections of STZ (dissolved in citrate buffer at pH 4.5, at a dose of 50 mg/kg body weight; Sigma–Aldrich) or citrate buffer for 5 days in a row. After 1 week, mice with blood glucose levels above 16.7 mmol/L were included in the study and continued on their diet for 12 more weeks. Mice were anesthetized with sodium pentobarbital (50 mg/kg, intraperitoneal injection; Sigma–Aldrich) and were euthanized via cervical dislocation under deep anesthesia at the study endpoint. Kidney tissues were collected prior to euthanizing the mice for additional experiments.

### 2.11. HE and Masson’s Trichrome Staining

We employed HE staining and Masson’s trichrome staining to evaluate the success of the DKD mouse model as previously reported [40]. HE staining is mainly used to observe the overall tissue structure and cellular morphology. Masson’s trichrome staining specifically detects collagen fibers, a key connective tissue component, enabling the assessment of fibrosis degree. For HE staining, mouse kidney tissues were fixed in 4% paraformaldehyde, dehydrated through graded ethanol, cleared in xylene, and embedded in paraffin. Then, 5‐μm‐thick sections were cut. The sections were deparaffinized, rehydrated, stained with hematoxylin for nuclei and eosin for cytoplasm, followed by dehydration and mounting. For Masson’s trichrome staining, we used the Masson’s Trichrome Stain Kit (Catalog No. D026‐1‐3, Nanjing Jiancheng). Sections were treated according to the kit instructions, staining collagen fibers blue and other components different colors for fibrosis assessment.

### 2.12. qPCR

To quantitatively detect the expression levels of specific genes in the kidneys of DKD mice, we employed real‐time fluorescent qPCR technology. Initially, we extracted total RNA from mouse kidney tissue and converted it into cDNA using reverse transcriptase. Subsequently, we designed specific primers targeting genes such as Colla2, Ptprc, Tyrobp, and Csf1r, and utilized these primers for qPCR amplification. Meanwhile, we selected β‐actin as the endogenous control gene and designed corresponding primers. During the amplification process, the fluorescent dye binds to the DNA double strands, emitting a fluorescent signal. By monitoring the real‐time changes in the intensity of the fluorescent signal, we calculated the relative expression of the mRNA using the 2^−ΔΔCt^ method for normalization. The PCR primer in Table 1. Statistical analysis was performed using Student’s *t*‐test to compare the relative expression levels of genes between different groups (number of replicates = 5).

**Table 1:** The PCR primer.

Gene	Forward primer	Reverse primer
Colla2	CTAGCCAACCGTGCTTCTCA	TCTCCTCATCCAGGTACGCA
Ptprc	TTGTCACAGGGCAAACACCT	GGGGTCACTTTGAGGCAGAA
Tyrobp	GATTGCCCTGGCTGTGTACT	TGTTGTTTCCGGGTCCCTTC
Csf1r	GGAGGTGACAGTGGTTGAGG	CCGTTTTGCGTAAGACCTGC
β‐actin	GGCTGTATTCCCCTCCATCG	CCAGTTGGTAACAATGCCATGT

### 2.13. Immunohistochemical Staining

Firstly, mouse kidney tissue sections underwent preprocessing steps including dewaxing, rehydration, and antigen retrieval. Subsequently, we incubated the sections with specific antibodies targeting the proteins of interest (COLLA2, PTPRC, TYROBP, and CSF1R), with all primary antibodies (all Sangon Biotech) diluted at a ratio of 1:1000, allowing the antibodies to bind to the target proteins in the sections. Next, we used secondary antibodies to bind to the primary antibodies and observed the intensity and distribution of chemiluminescent signals through a microscope, thereby assessing the expression levels of the target proteins in the kidney tissues.

### 2.14. Evaluation Method for Immune Cell Infiltration

To quantify the relative abundances of immune cells, we employed the CIBERSORT deconvolution algorithm [41] using the standard LM22 signature matrix, which defines 22 human immune cell phenotypes (LM22 was obtained from the CIBERSORTx website https://cibersortx.stanford.edu/). The analysis was performed in signature matrix mode, which outputs the relative proportion of each cell type within the total inferred immune cell compartment. The algorithm’s default settings were used without quantile normalization, as our analysis was conducted on a single dataset. To ensure the reliability of the deconvolution results, we applied a stringent quality control filter: only samples with a CIBERSORT‐calculated empirical *p*‐value < 0.05 were retained for all subsequent statistical analyses and visualizations. The ggplot2 package was employed to create bar charts illustrating the immune abundance in the normal versus control groups [42] was used for visualization. Additionally, the Spearman statistical method was used to analyze the correlations among them individually. Finally, box plots, clustered stacked bar charts, lollipop plots, correlation scatter plots, and correlation network heatmaps were drawn to present the results of the immune infiltration analysis. The linkET package was used for the calculation of the correlation network heatmap data, and ggplot2 was used for visualization.

### 2.15. SnRNA‐Seq Data Processing

snRNA‐seq expression profile data (GSE131882) was obtained from the GEO database (website: http://www.ncbi.nlm.nih.gov/) [43]. We screened eligible samples, including 3 diabetic kidney tissue samples and 3 normal kidney tissue samples. Data analysis was conducted using the Seurat package [44] (version 5.1.0). Firstly, DoubletFinder [45] was used to identify doublet cells from each sample. Doublets were filtered out, and metrics including nFeature_RNA, nCount_RNA, and percent_mito were calculated for each sample. Thresholds were selected based on violin plots (Supporting Information 2: Figure S1) to filter out cells with apparent outliers. Cells meeting the following criteria were retained: nFeature_RNA > 500 and < 3000; nCount_RNA < 6000; percent_mito < 5. Cells were merged with Seurat’s merge function and analyzed using the NormalizeData function (LogNormalize method, scale.factor = 10,000). Top 2000 variable features were identified via FindVariableFeatures, followed by PCA dimensionality reduction (RunPCA, npcs = 50). Batch effects were corrected with RunHarmony [46, 47] followed by nonlinear dimensionality reduction using RunUMAP. To assess the efficacy of batch effect removal, we employed the k‐nearest neighbor batch effect test (kBET). FindNeighbors constructed the neighbor graph (k.param = 30) with top 20 principal components. Cell clusters were generated via FindClusters (resolution = 0.8), and cluster markers identified by FindAllMarkers (min.pct = 0.6, logfc.threshold = 1, test.use = "MAST").

Using FindAllMarkers, we identified cluster‐specific marker genes and annotated kidney tissue cell clusters via classic marker gene expression: principal cells (AQP2), thick ascending limbs of Henle’s loop (SLC12A1), distal convoluted tubules (SLC12A3), proximal convoluted tubules (PCTs) (SLC5A12, SLC22A8, SLC17A3), proximal tubules (MIOX, GPX3), connecting tubules (SLC8A1), intercalated cells type A (SLC26A7), and podocytes (KLF6).

Furthermore, we integrated known lineage markers with the human placental cell atlas (CellMarker; http://xteam.xbio.top/CellMarker/) for manual annotation [48], ensuring accuracy. DEGs were identified via Wilcoxon rank‐sum test (FindMarkers in Seurat, *p*_val <0.05, |avg_log2FC|>0.5) and visualized with ggplot.

### 2.16. Cell–Cell Communication

We applied CellChat R package (v1.5.0) [49] to analyze intercellular communication through ligand‐receptor‐cofactor interactions. Firstly, a random seed (seed = 0528) was set, and 6000 cells were randomly selected, with all cells from DKD samples extracted to create a CellChat object. Subsequently, cell–cell interaction networks were visualized via netVisual_circle (interaction number/intensity). Bubble and hierarchy plots (netVisual_bubble/netVisual_hierarchy) highlighted ligand–receptor interactions between target and other clusters. NMF [50] revealed communication patterns. The NMF package (version 0.27) was used to perform non‐negative matrix factorization (NMF) on the CellChat object, and the selectK function was employed to identify the best number of patterns and to group cells with similar communication behaviors. Finally, we conducted network centrality analysis on different cell clusters to uncover the possible roles of each cell in the communication network [51].

### 2.17. Pseudo‐Temporal Analysis

This study employed the “Monocle” package (version 2.32.0) for unsupervised pseudotime analysis [52]. Firstly, the newCellDataSet function, with the parameter expression Family = negbinomial.size, was used to construct a cell dataset containing the expression matrix, phenotypic data, and feature data. Next, the estimateSizeFactors and estimateDispersions functions were used to correct the scale factors and dispersion of gene expression among cells. Subsequently, the DDRTree method (with the parameter max_components = 2) was employed to reduce dimensions [53], and the plot_cell_trajectory function was used to sort and visualize the cells. Highly variable pseudotime‐associated genes were screened and analyzed for expression dynamics using differentialGeneTest. Expression patterns were clustered (plot_pseudotime_heatmap) and visualized. BEAM analysis [54] identified branching genes with elevated thresholds. BEAM genes were visualized via plot_genes_branched_heatmap. KEGG enrichment analysis (clusterProfiler v4.12.0, org.Hs.eg.db v3.19.1) was performed on cluster genes, with results visualized via ggplot2.

## 3. Results

### 3.1. Screening of Co‐Expressed DEGs (Co‐DEGs)

The datasets GSE142025 and Merged_GSE47183_GSE32591 were normalized (Figure 1A,B). PCA revealed distinct clustering results for both datasets. In the GSE142025 dataset, PC1 accounted for 29.2% and PC2 for 15.8% of the variance (Figure 1C). For the Merged_GSE47183_GSE32591 dataset, PC1 was 23.3% and PC2 was 11.3% (Figure 1D), indicating significant differences between groups, and the reduced‐dimension coordinates of PC1 and PC2 for each sample can be found in Supporting Information 3: File S2. Volcano plots, using |log2(FC)| > 1 and Padj. < 0.05 as screening thresholds, identified 1059 DEGs in the GSE142025 dataset, a total of 636 genes experienced up‐regulation, whereas 423 genes experienced down‐regulation (Figure 1E). Applying the same thresholds, 598 DEGs were identified in the Merged_GSE47183_GSE32591 dataset, comprising 262 genes with increased expression and 336 genes with decreased expression (Figure 1F). The complete ranked list is provided in Supporting Information 4: File S3. Heatmaps illustrate the top 20 genes with increased and decreased expression in each dataset separately (Figure 1G,H).

Figure 1Screening of co‐expressed DEGs. (A, B) Boxplots of GSE142025 and Merged_GSE47183_GSE32591 datasets after normalization. (C, D) PCA of GSE142025 and Merged_GSE47183_GSE32591 datasets. (E, F) Volcano plots of differentially expressed mRNAs in GSE142025 and Merged_GSE47183_GSE32591. (G, H) Heatmaps showing the genes with increased and decreased expression in GSE142025 and Merged_GSE47183_GSE32591 datasets, respectively.

**Figure:**
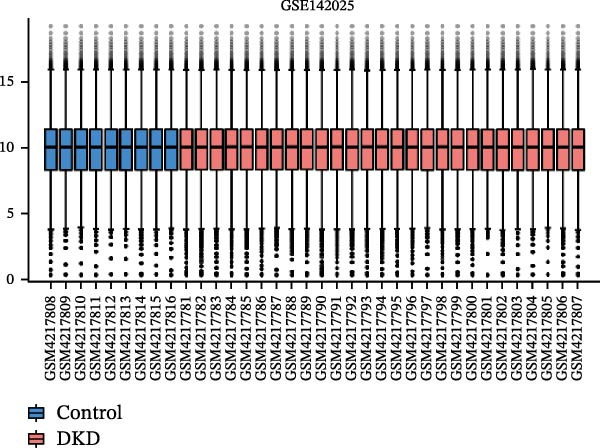
(A)

**Figure:**
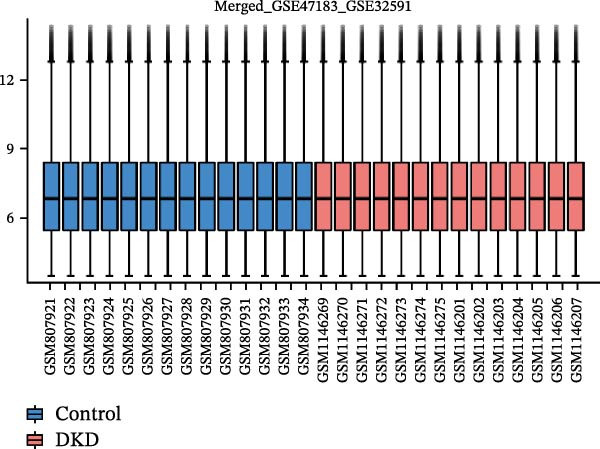
(B)

**Figure:**
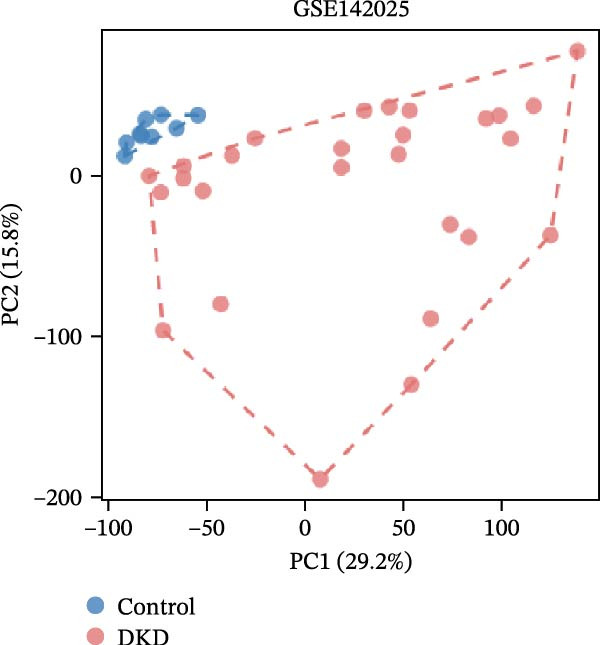
(C)

**Figure:**
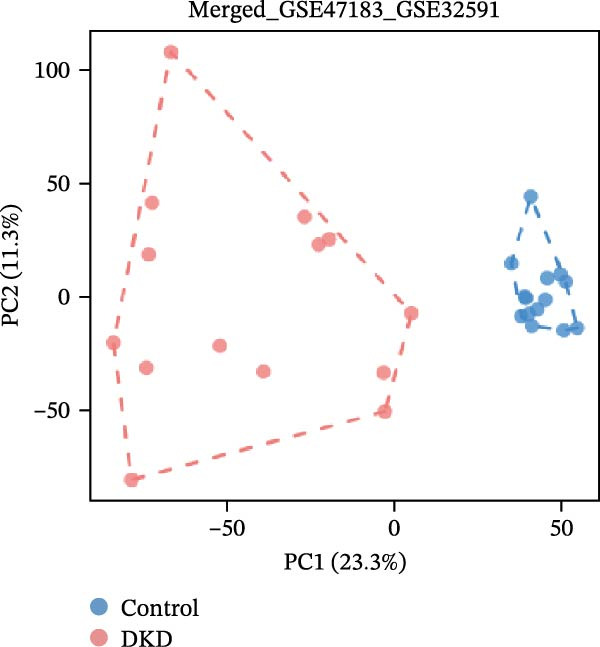
(D)

**Figure:**
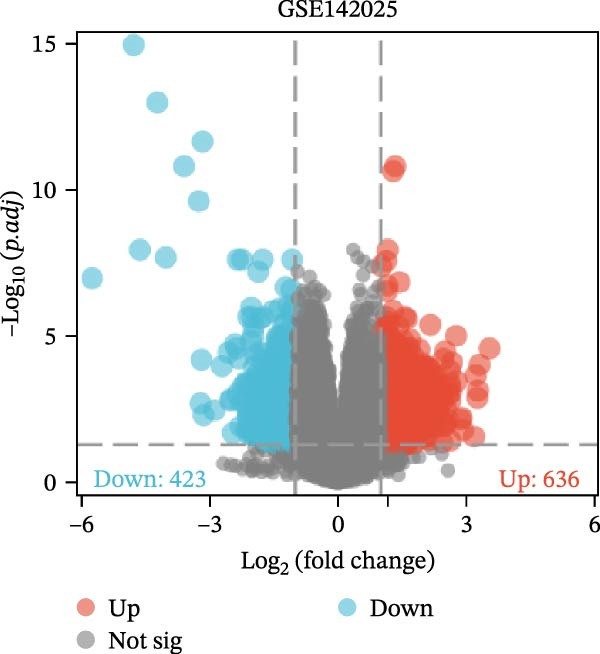
(E)

**Figure:**
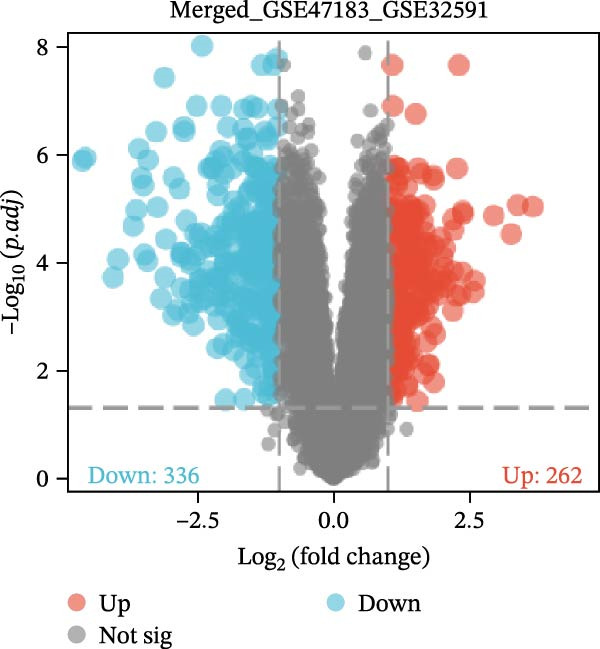
(F)

**Figure:**
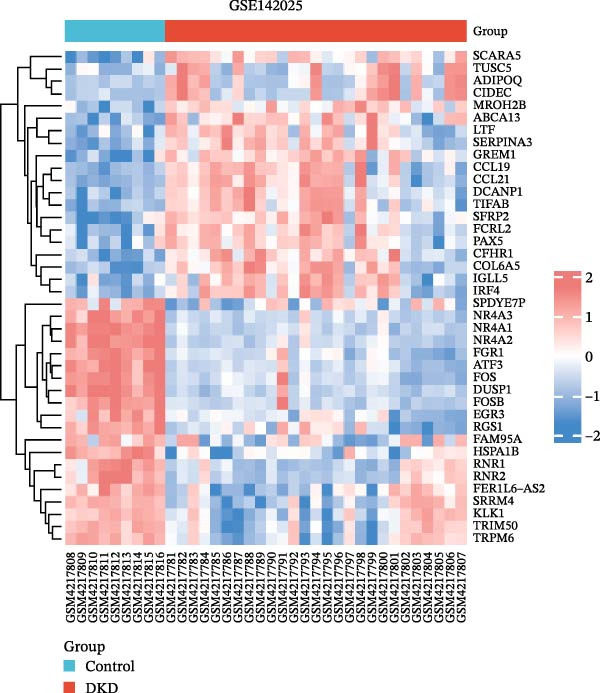
(G)

**Figure:**
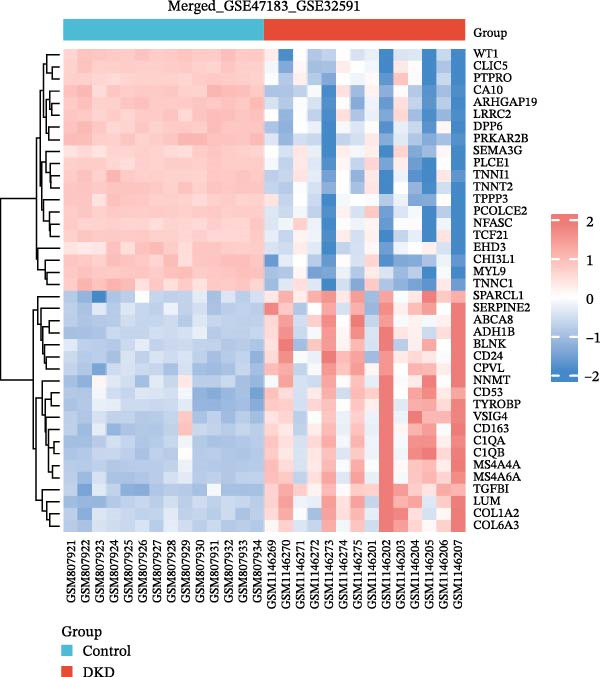
(H)

### 3.2. GO and KEGG Enrichment Analysis of Co‐DEGs

The DEGs obtained from GSE142025 and Merged_GSE47183_GSE32591, respectively, were intersected with autophagy‐related genes, resulting in 49 genes (Figure 2A). Functional enrichment analysis of GO terms and KEGG signaling pathways was conducted for the 49 Co‐DEGs (Figure 2B). These DEGs were significantly enriched in BP such as myeloid leukocyte activation, positive regulation of cytokine production, collagen‐containing extracellular matrix, heparin binding, glycosaminoglycan binding (Figure 2C). The DEGs were primarily involved in pathways related to diabetic complications, including PI3K‐Akt signaling pathway, MAPK signaling pathway, AGE‐RAGE signaling pathway, osteoclast differentiation, and ECM–receptor interaction (Figure 2D).

Figure 2Functional enrichment analysis of GO/KEGG for the 49 Co‐DEGs. (A) Venn diagram of the 49 Co‐DEGs. (B) GO and KEGG enrichment analysis. GO analysis encompasses BP, CC, and MF. (C) Circular visualization of GO enrichment analysis. (D) Chord diagram visualization of the 12 significantly enriched KEGG pathways.

**Figure:**
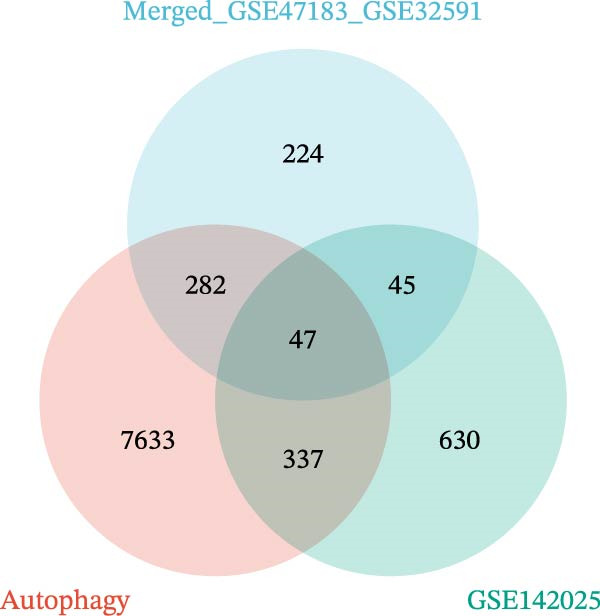
(A)

**Figure:**
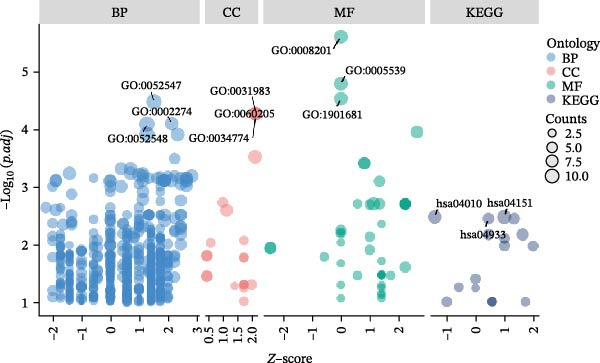
(B)

**Figure:**
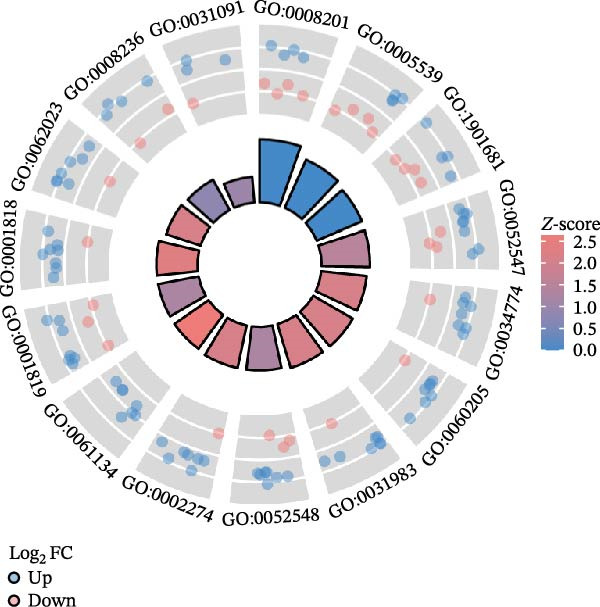
(C)

**Figure:**
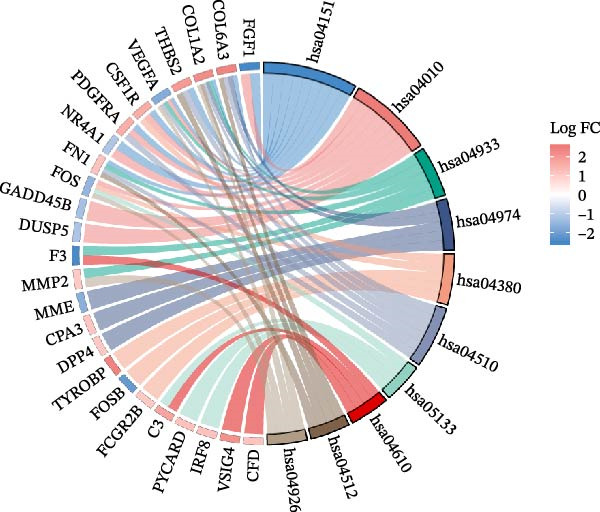
(D)

### 3.3. PPI Network

Subsequently, the STRING database was utilized for analysis to construct a PPI network for 49 genes (Figure 3A). The CytoHubba plugin was employed to identify the top 20 hub genes based on the MNC, Betweenness, MCC, EPC, and Degree algorithms (Figure 3B–F). Next, the results from these five algorithms were intersected to obtain 11 hub genes (Figure 3G). Finally, we also investigated the chromosomal locations of these 11 hub genes (Figure 3H) and their correlations (Figure 3I).

Figure 3PPI network and 11 hub genes. (A) PPI network. Nodes represent proteins, and edges indicate interactions between proteins. The intensity of node color indicates the degree metric, representing its numerical value and importance in the network. (B–F) Top 20 genes obtained through the MNC, Betweenness, MCC, EPC, and Degree algorithms. (G) Venn diagram showing the 11 hub genes selected by crossing the results of five CytoHubba algorithms. (H) Chromosomal locations of the 11 key genes. (I) Correlation heatmap: used to identify correlations among the 11 hub genes.

**Figure:**
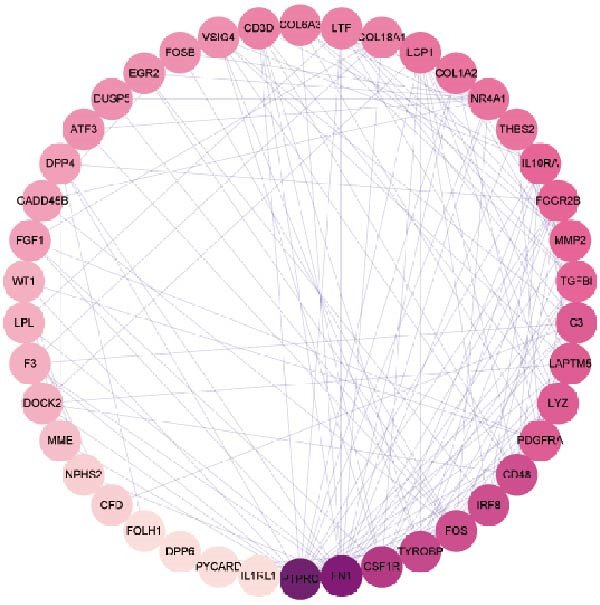
(A)

**Figure:**
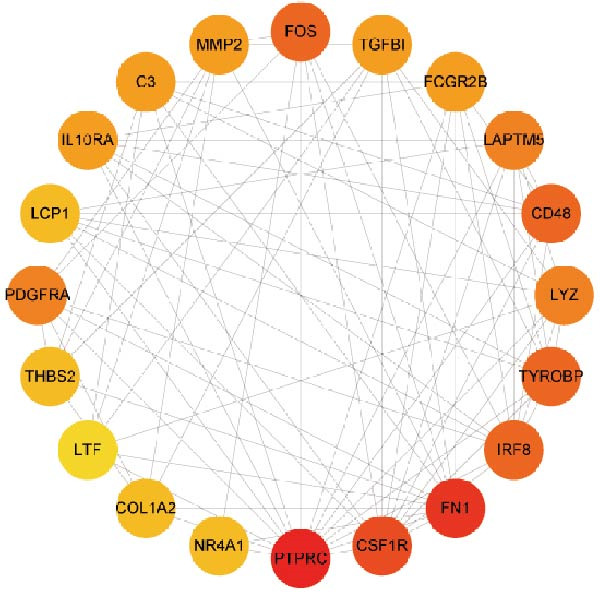
(B)

**Figure:**
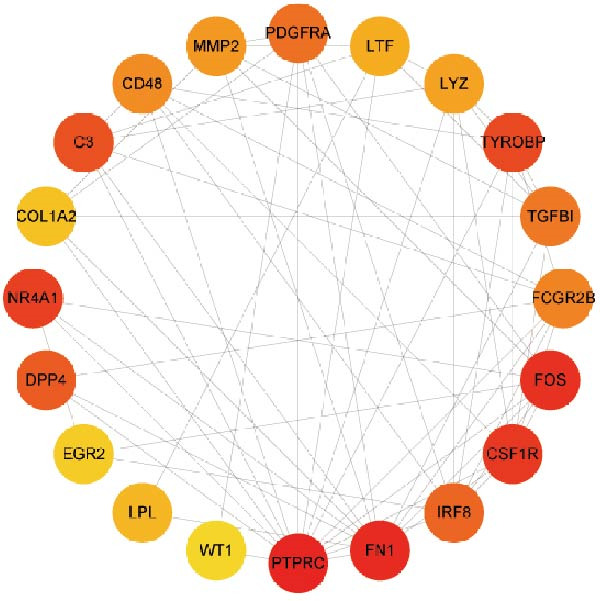
(C)

**Figure:**
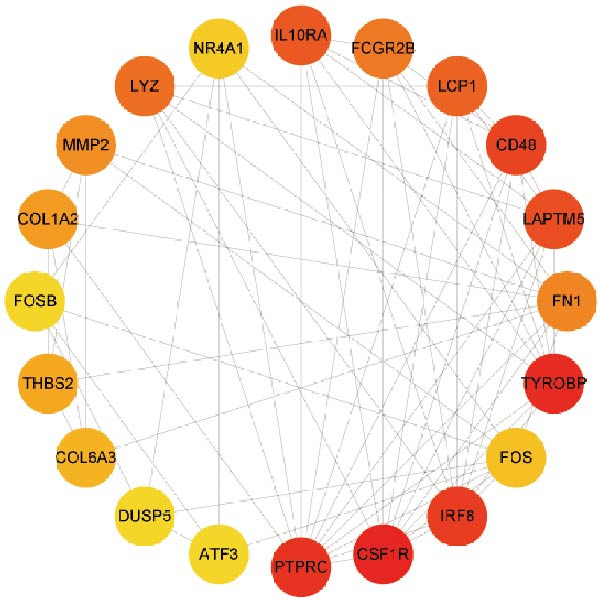
(D)

**Figure:**
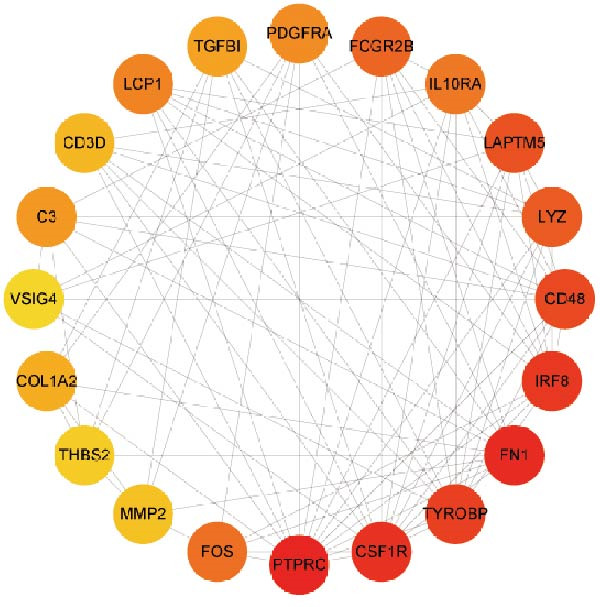
(E)

**Figure:**
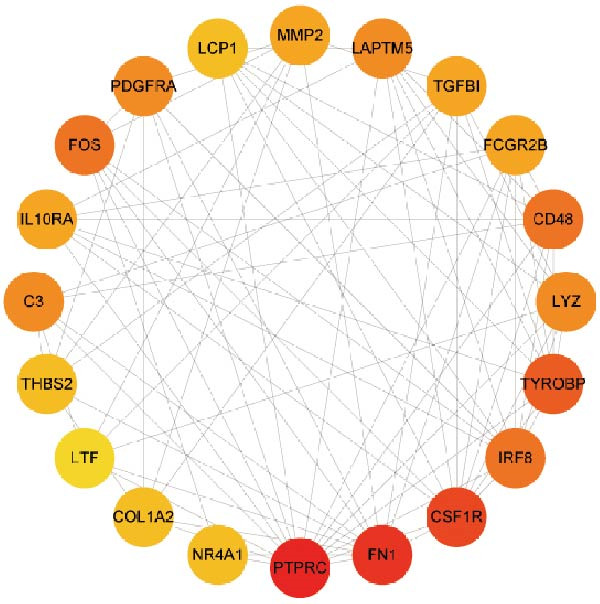
(F)

**Figure:**
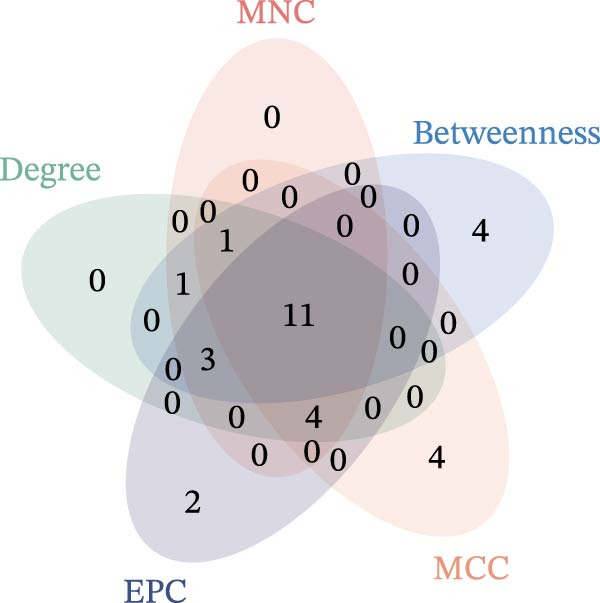
(G)

**Figure:**
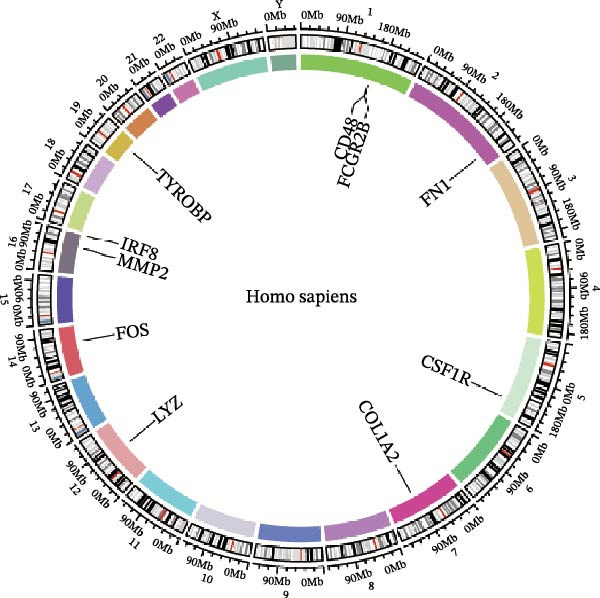
(H)

**Figure:**
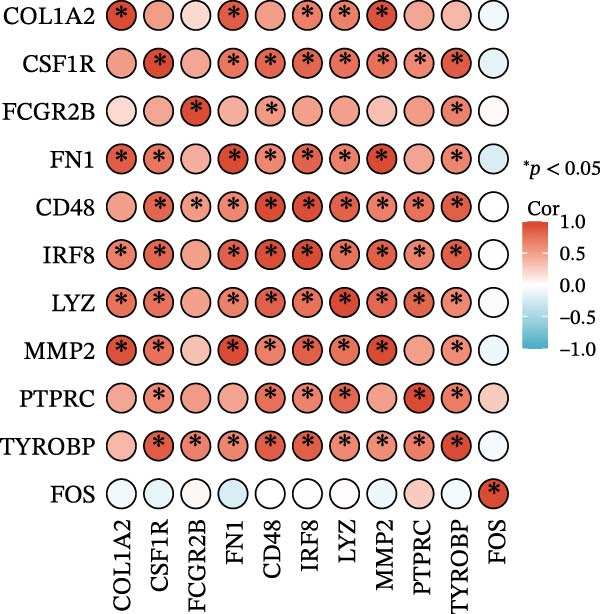
(I)

### 3.4. Identification of Potential Diagnostic Biomarkers for DKD Using Machine Learning

We employed LASSO, RF, SVM, and LDA to screen hub genes, respectively. Through LASSO, we obtained five important variables, including COL1A2, CSF1R, FOS, PTPRC, and TYROBP (Figure 4A,B). For the RF algorithm, with an average Gini index decrease >1 as the screening criterion, five important variables were selected: TYROBP, CSF1R, PTPRC, COL1A2, and MMP2 (Figure 4C). Using SVM, seven features were identified, including CSF1R, TYROBP, PTPRC, COL1A2, MMP2, FN1, and LYZ (Figure 4D). The LDA algorithm screened out nine features: MMP2, CSF1R, COL1A2, PTPRC, TYROBP, FN1, LYZ, IRF8, and CD48 (Figure 4E). By intersecting the characteristic genes obtained from the four machine learning algorithms, we ultimately identified four potential diagnostic biomarkers for DKD: COL1A2, CSF1R, PTPRC, and TYROBP. The results are presented in a Venn diagram (Figure 4F).

Figure 4Further screening of optimal diagnostic gene biomarkers for DKD using machine learning. (A, B) Five characteristic genes were identified through the LASSO algorithm. A shows the LASSO variable trajectory visualization, and B shows the LASSO coefficient selection process visualization. (C) Five feature genes were screened using the Random Forest algorithm with the mean decrease in Gini index as the metric. (D) Seven feature genes were selected through Recursive Feature Elimination with the SVM algorithm, choosing the set where the model error rate was lowest. (E) Feature genes included in the model with the highest accuracy were selected using the LDA algorithm, with model accuracy as the metric. (F) Venn diagram showing the intersection of results from the four machine learning algorithms, obtaining DKD biomarkers recognized by all four methods.

**Figure:**
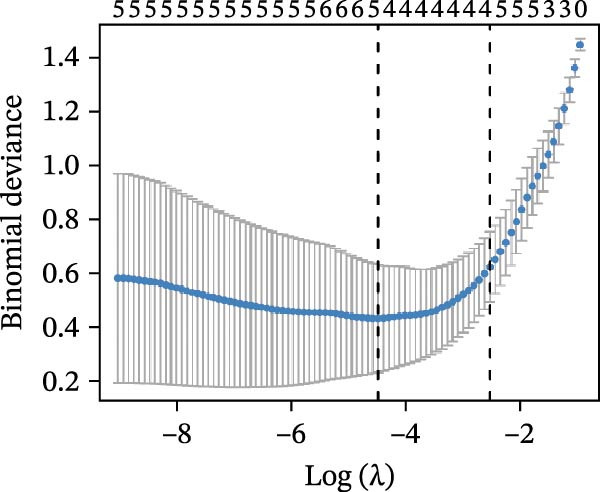
(A)

**Figure:**
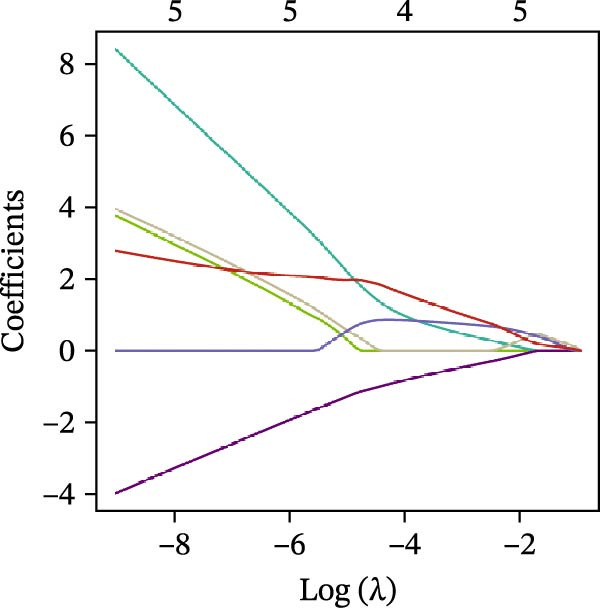
(B)

**Figure:**
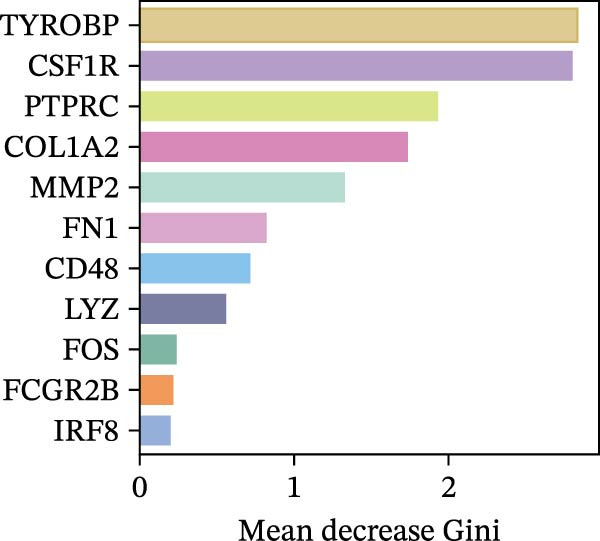
(C)

**Figure:**
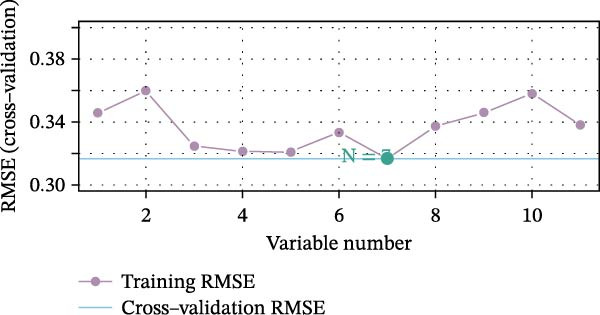
(D)

**Figure:**
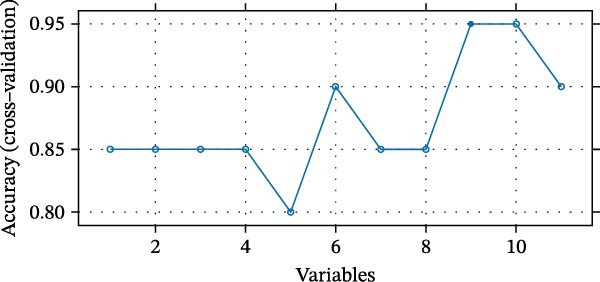
(E)

**Figure:**
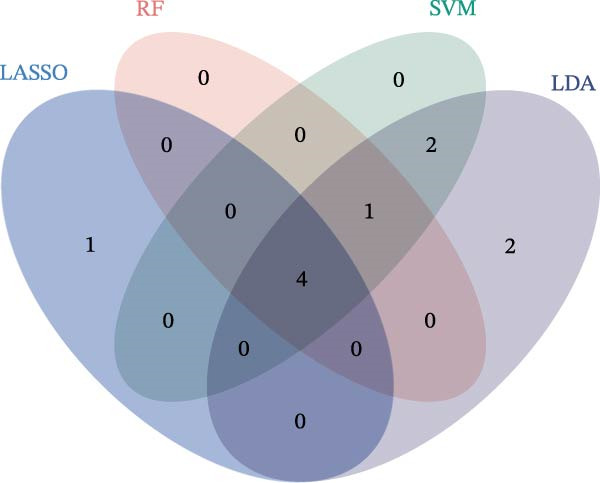
(F)

### 3.5. Expression Profiles and Identification of Potential Diagnostic Biomarkers for DKD

We used datasets GSE142025, Merged_GSE47183_GSE32591, and GSE104948 to examine the expression profiles of the four potential diagnostic biomarkers for DKD. The results showed that when Merged_GSE47183_GSE32591 was used as the training set, the expression levels of COL1A2, CSF1R, PTPRC, and TYROBP were all higher in the DKD (Figure 5A–D). ROC curves were employed to evaluate the diagnostic performance of these four genes in DKD. The results indicated that in the training set Merged_GSE47183_GSE32591, the AUCs for COL1A2, CSF1R, PTPRC, and TYROBP were all above 0.9 (Figure 5E‐H), demonstrating high predictive accuracy. In the training set GSE142025, the AUCs for COL1A2, CSF1R, PTPRC, and TYROBP were all above 0.7 (Figure 5I–L), also showing high predictive accuracy. Subsequently, we performed external validation using the independent dataset GSE104948, and the results showed that the AUCs for COL1A2, CSF1R, PTPRC, and TYROBP were all above 0.9, consistent with the predictive results (Figure 5M–P), Detailed statistical information can be found in Supporting Information 5: File S4. Finally, four genes, COL1A2, CSF1R, PTPRC, and TYROBP, were selected as potential diagnostic biomarkers related to autophagy in DKD.

Figure 5Identification of diagnostic biomarkers. (A–D) Analysis of the expression levels of 4 DKD genes in DKD and healthy samples using the Merged_GSE47183_GSE32591 dataset ( ^∗^ *p*  < 0.05;  ^∗∗^ *p*  < 0.01;  ^∗∗∗^ *p*  < 0.001). (E–H) ROC curves for the expression of four genes for DKD in Merged_GSE47183_GSE32591. (I–L) ROC curves for the expression of 4 genes for DKD in GSE142025. (M–P) ROC curves for the model in the GSE104948 validation set.

**Figure:**
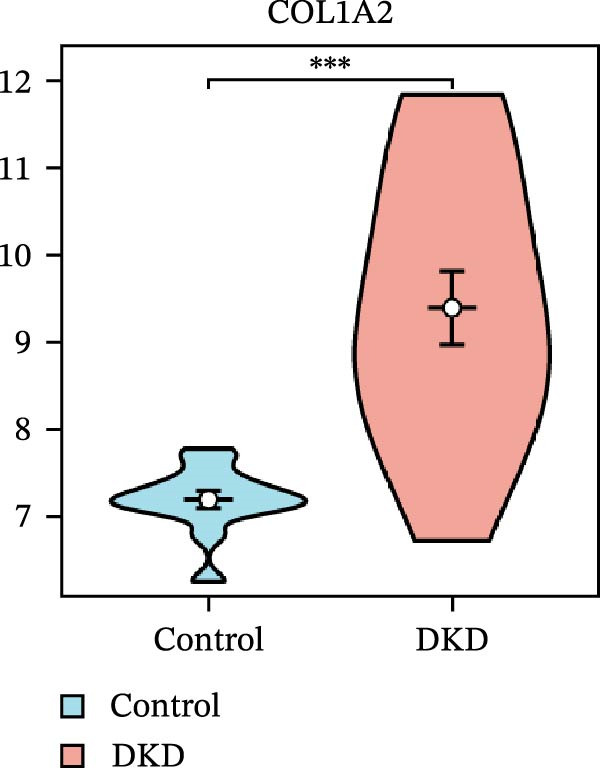
(A)

**Figure:**
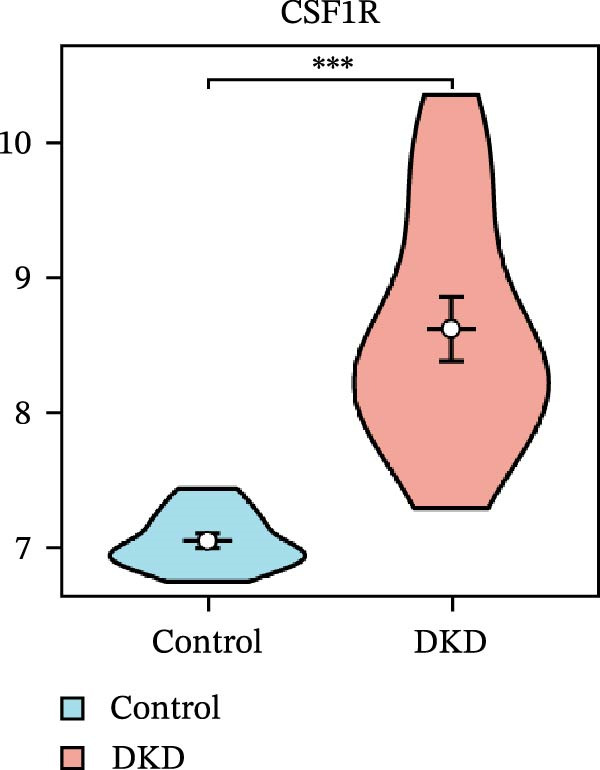
(B)

**Figure:**
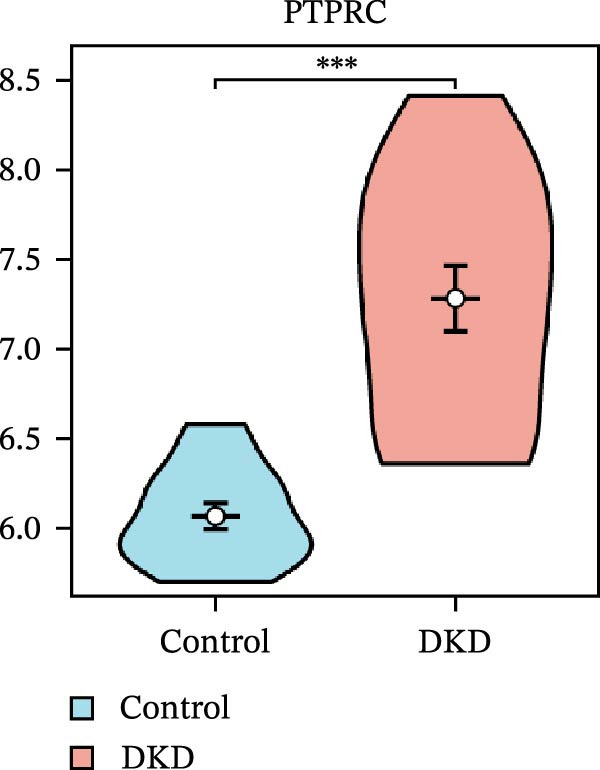
(C)

**Figure:**
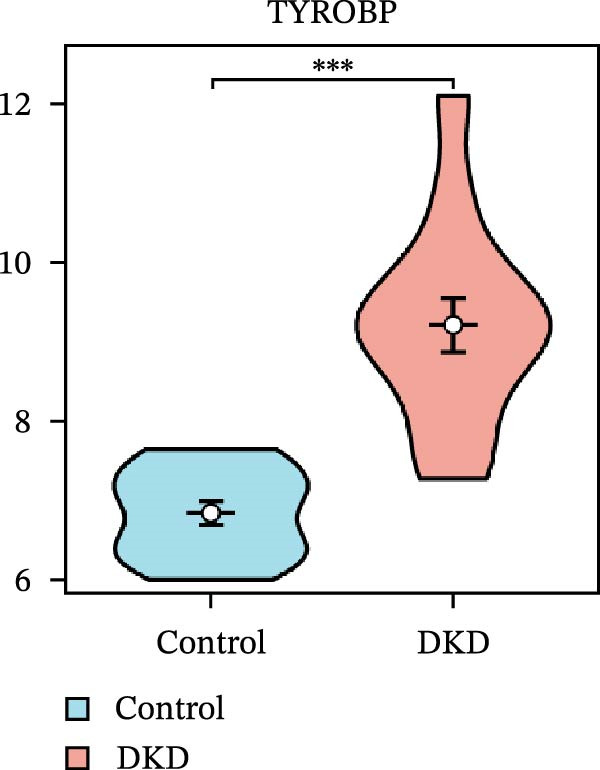
(D)

**Figure:**
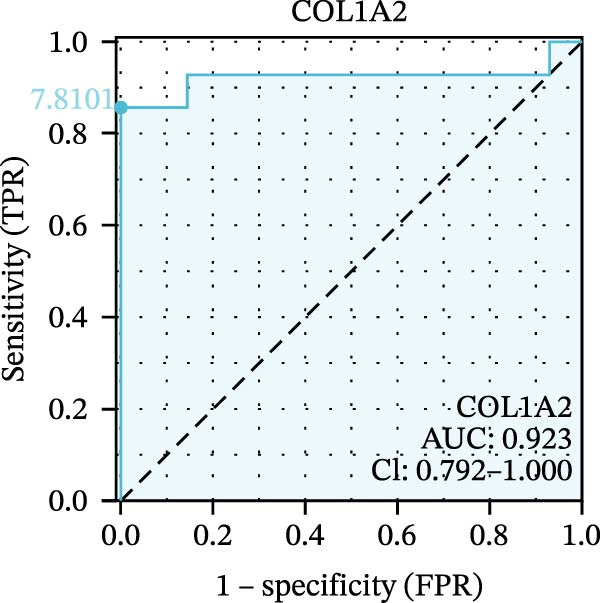
(E)

**Figure:**
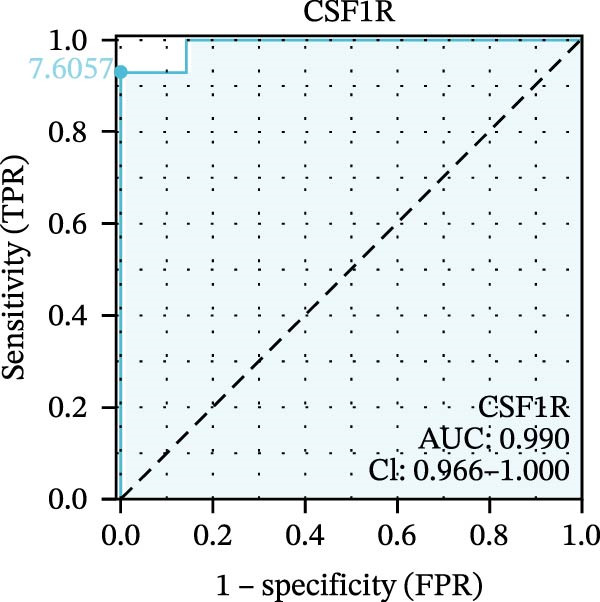
(F)

**Figure:**
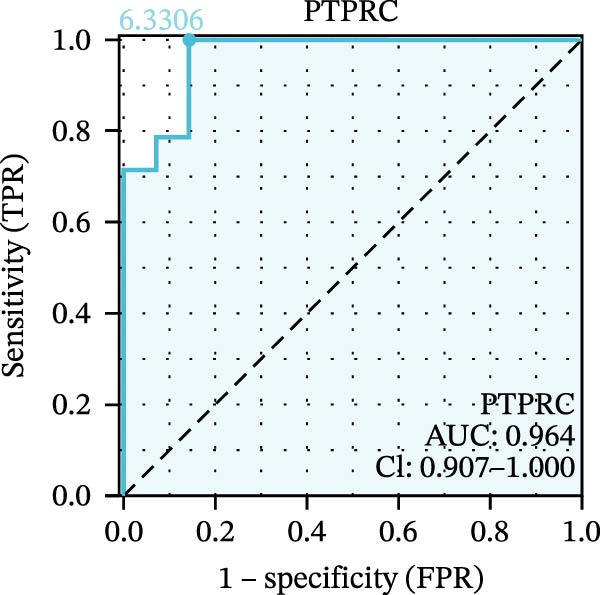
(G)

**Figure:**
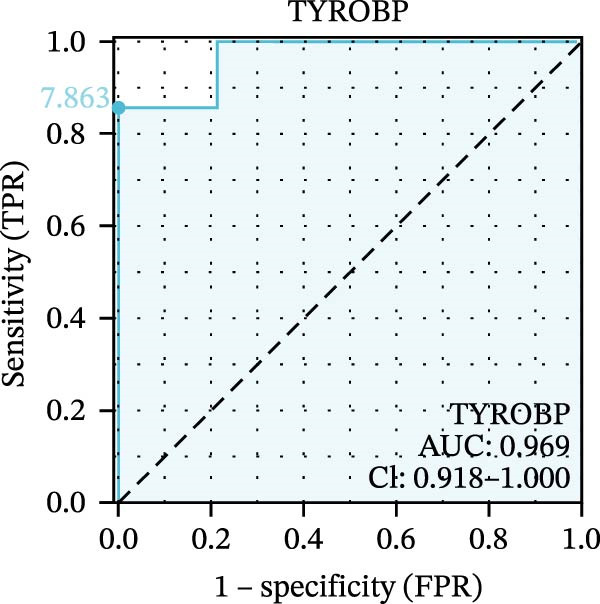
(H)

**Figure:**
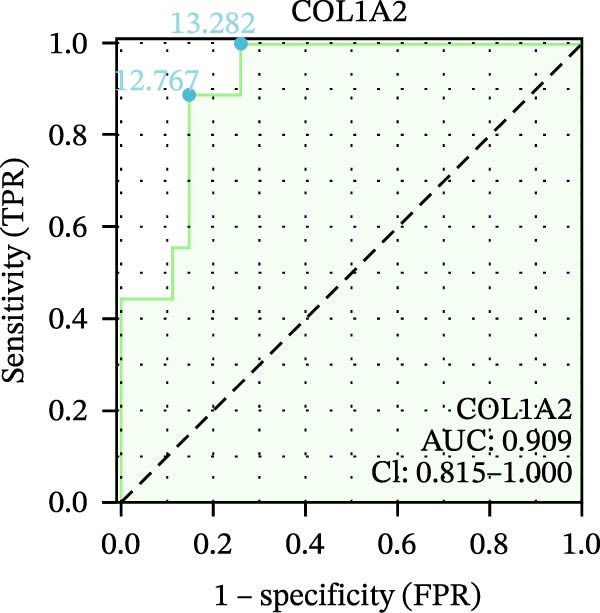
(I)

**Figure:**
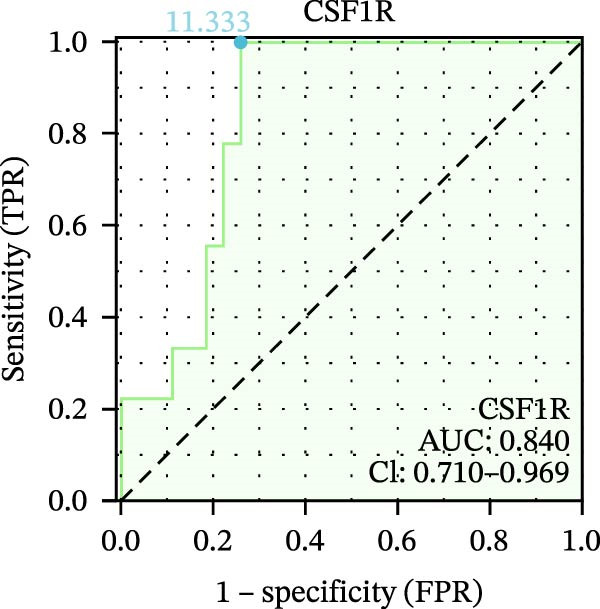
(J)

**Figure:**
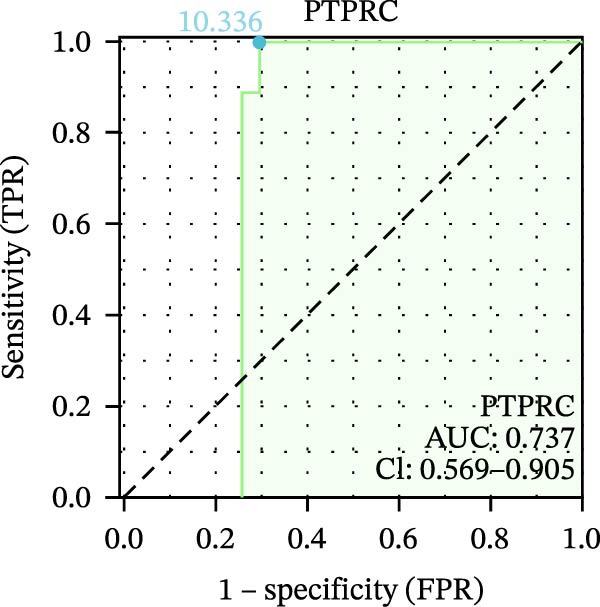
(K)

**Figure:**
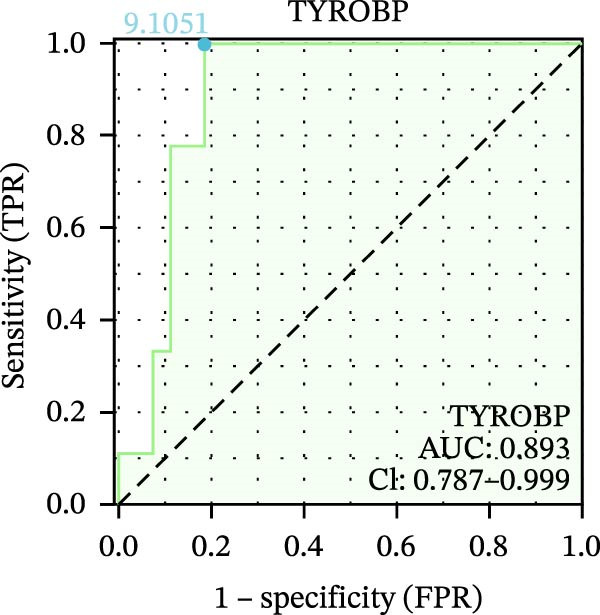
(L)

**Figure:**
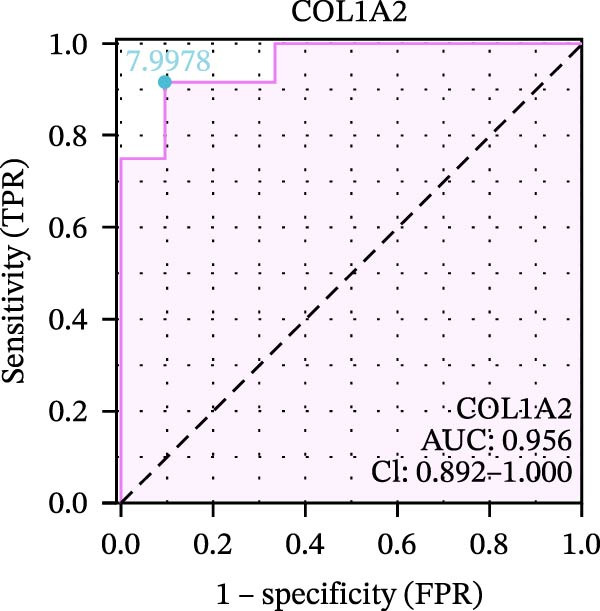
(M)

**Figure:**
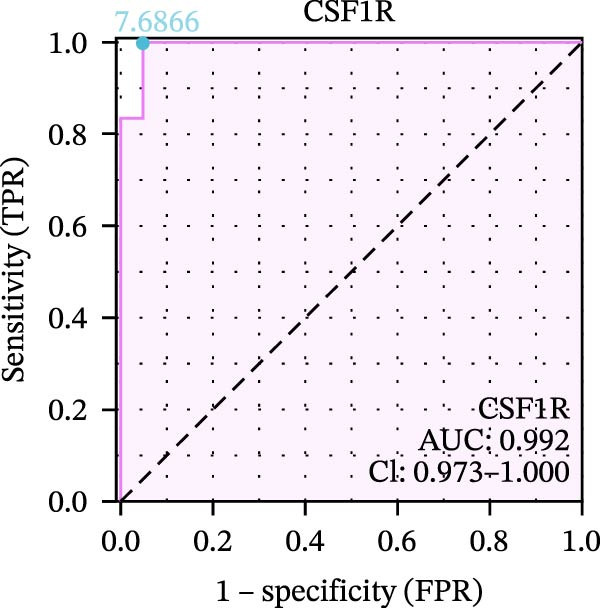
(N)

**Figure:**
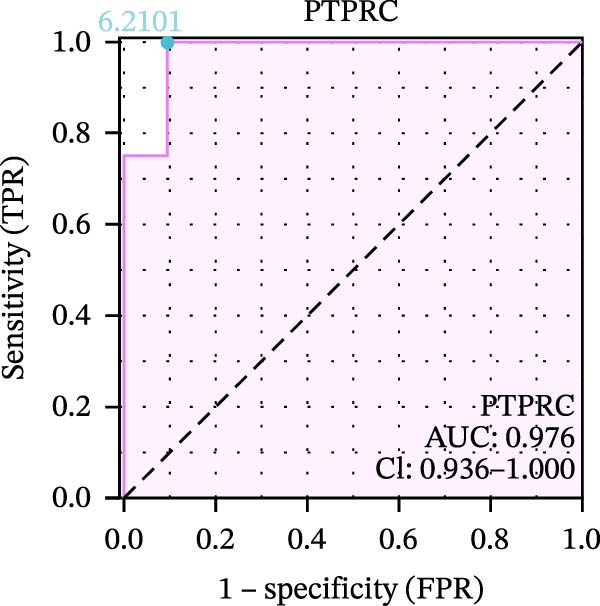
(O)

**Figure:**
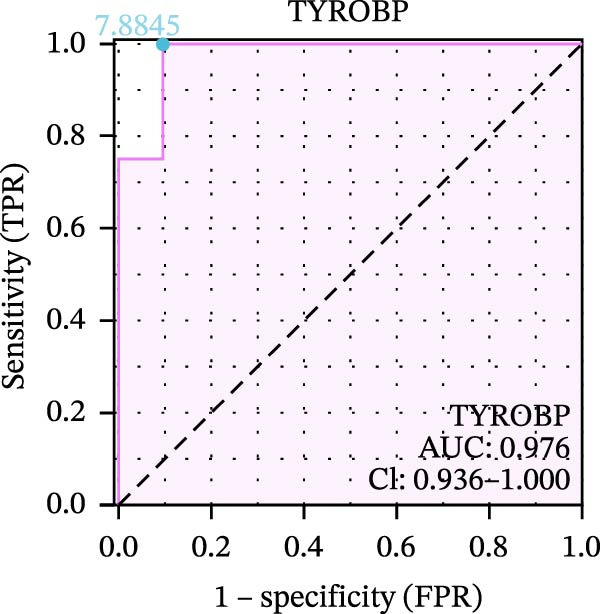
(P)

In the GSEA enrichment analysis, COL1A2 and CSF1R were co‐enriched in the PI3K‐AKT signaling pathway and focal adhesion‐PI3K‐AKT‐mTOR signaling pathway (Figure 6B,C). PTPRC and TYROBP were co‐enriched in Reactome neutrophil degranulation (Figure 6D). Additionally, PTPRC was specifically enriched in the T‐cell receptor signaling pathway (Figure 6E).

Figure 6Enrichment analysis. (A) Distribution of potential diagnostic biomarkers in the KEGG enrichment analysis. (B–E) Visualization of GSEA results associated with potential diagnostic biomarkers.

**Figure:**
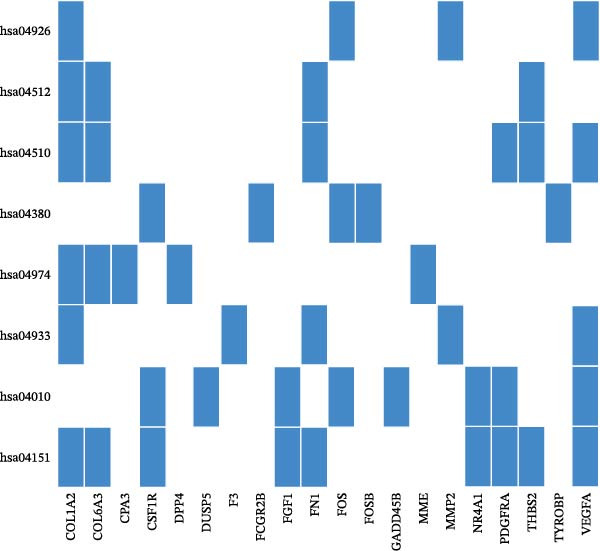
(A)

**Figure:**
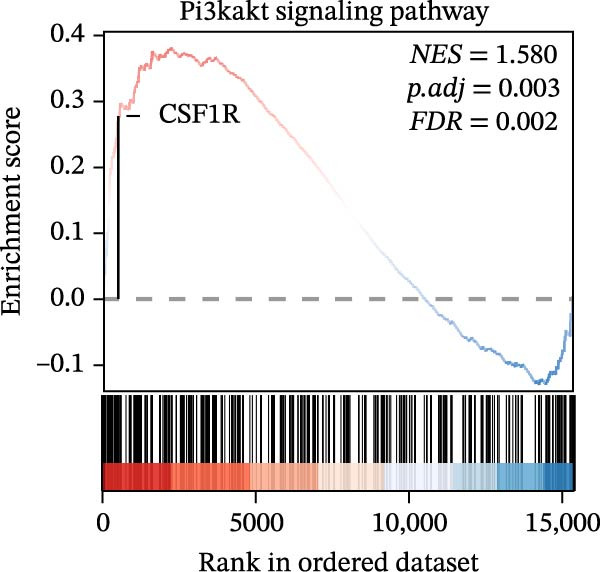
(B)

**Figure:**
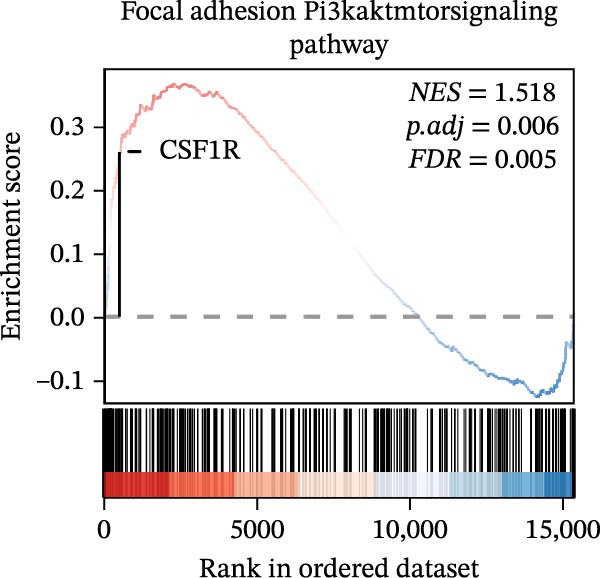
(C)

**Figure:**
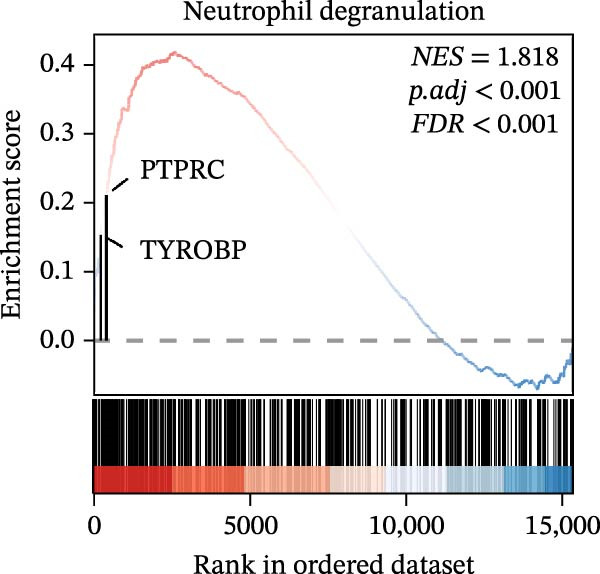
(D)

**Figure:**
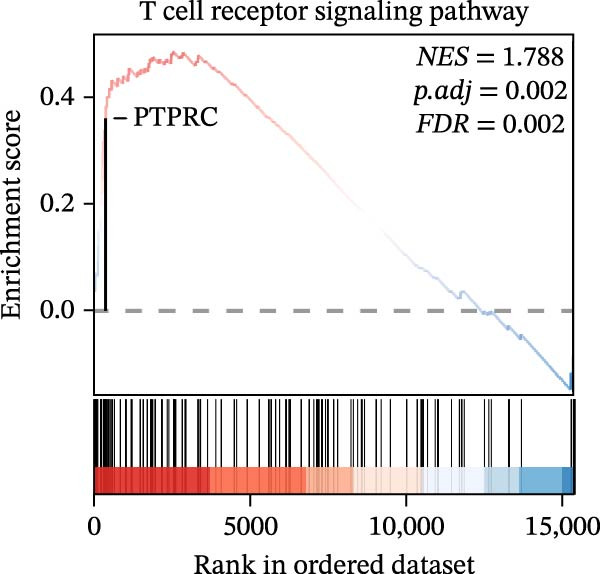
(E)

In the KEGG enrichment analysis, COL1A2 and CSF1R were enriched in the PI3K‐Akt signaling pathway; CSF1R and TYROBP were primarily enriched in osteoclast differentiation. COL1A2 was significantly enriched in AGE‐RAGE signaling pathway in diabetic complications; protein digestion and absorption; focal adhesion; ECM–receptor interaction and relaxin signaling pathway. CSF1R showed enrichment in MAPK signaling pathway, and osteoclast differentiation (Figure 6A).

To verify the expression profile of key genes, we established a mouse model of DKD. Histological examination using HE and Masson’s trichrome staining (Figure 7A) verified the successful creation of the mouse model, demonstrating evident fibrotic phenotypes in DKD mice. Masson’s trichrome staining confirms the presence of evident fibrotic phenotypes in the DKD mouse model, indicating increased collagen deposition and fibrosis within the kidney tissue. Control mice show minimal collagen staining, in contrast to the DKD mice. qPCR analysis clearly showed an important elevation in the levels of Colla2, Ptprc, Tyrobp, and Csf1r expression (Figure 7B). Immunohistochemical staining (Figure 7C) further confirmed the marked upregulation of these key genes, aligning with the results obtained from bioinformatics analysis.

Figure 7Identification of diagnostic biomarkers for DKD (A) HE and Masson’s trichrome staining of kidney in control and DKD mice. (B) Results of qPCR analysis of 4 biomarker gene expression in the kidney tissues of DKD and control mice. (C) Immunohistochemical staining of kidney sections from DKD and control mice for biomarker gene. Values are presented as mean ± SD,  ^∗∗∗∗^ *p* < 0.001.

**Figure:**
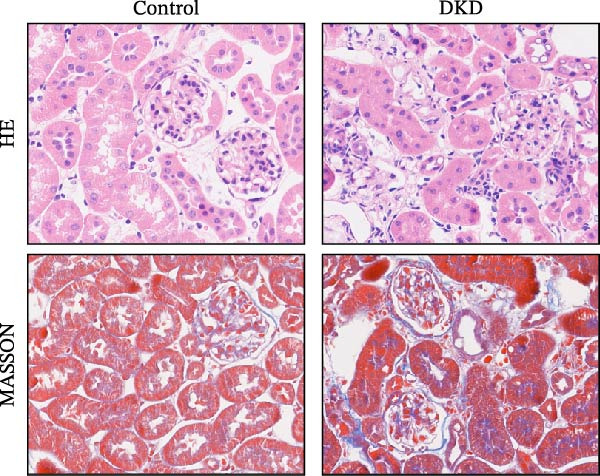
(A)

**Figure:**
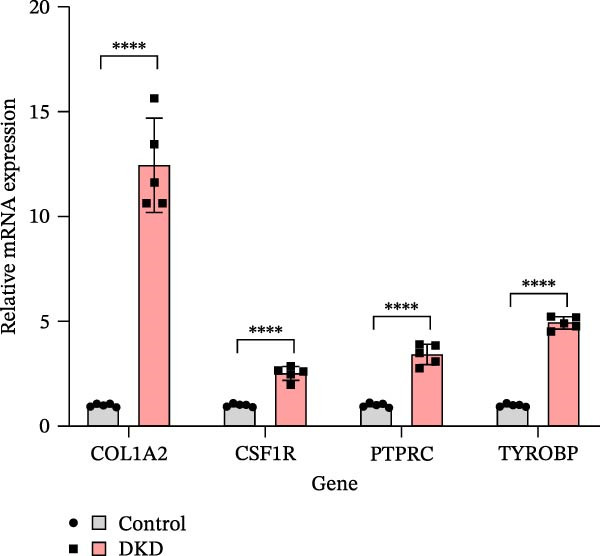
(B)

**Figure:**
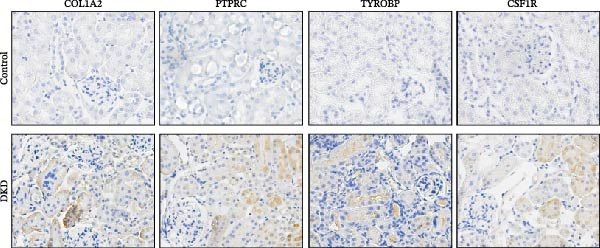
(C)

### 3.6. Immune Cell Infiltration Analysis

We employed the CIBERSORT method to evaluate the differences in immune cell infiltration and function between the disease and control groups (Figure 8A). The stacked bar plot displayed the relative abundance of various immune cells across different samples (Figure 8B). During correlation analysis, a positive correlation coefficient reflects a positive relationship between two variables, while a negative coefficient reflects a negative relationship. A weak correlation is represented by a correlation coefficient absolute value of 0.3–0.5, a moderate correlation by 0.5–0.8, and a strong correlation by 0.8–1. A statistically significant *p*‐value is one that is less than 0.05. Consequently, in DKD cases, the infiltration levels of resting Msat cells and regulatory T cells (Tregs) were positively associated with COL1A2 expression (*R* = 0.609, 0.660) (Figure 8C, Supporting Information 6: Figure S2A,C). The infiltration level of CD8 T cells was negatively correlated with COL1A2 expression (*R* = −0.727) (Figure 8C, Supporting Information 6: Figure S2B). There was a negative correlation between CSF1R expression and the infiltration levels of naïve B cells and resting NK cells (*R* = −0.591, −0.702) (Figure 8D, Supporting Information 6: Figure S2D, E). There was a positive correlation between the infiltration level of gamma delta T cells and PTPRC expression (*R* = 0.616) (Figure 8E, Supporting Information 6: Figure S2G), while an inverse relationship was observed between the infiltration level of resting NK cells and PTPRC expression (*R* = −0.614) (Figure 8E, Supporting Information 6: Figure S2F). There was a positive correlation between the infiltration level of gamma delta T cells and TYROBP expression (*R* = 0.680) (Figure 8F, Supporting Information 6: Figure S2J), whereas a negative correlation was observed between TYROBP expression and the infiltration levels of naïve B cells and resting NK cells (*R* = −0.780, −0.691) (Figure 8F, Supporting Information 6: Figure S2H, I). Within immune cells, there is also correlation among different types of immune cells (Supporting Information 6: Figure S2K).

Figure 8Immune cell infiltration assessment. (A) Box plots showing the differences in immune cell infiltration levels between the control and disease groups calculated by the cibersort method. (B) Stacked bar plots showing the infiltration proportions of various immune cells across all samples. (C–F) Lollipop plots illustrating the correlations between COL1A2 (C), CSF1R (D), PTPRC (E), and TYROBP (F) with immune cells, respectively.

**Figure:**
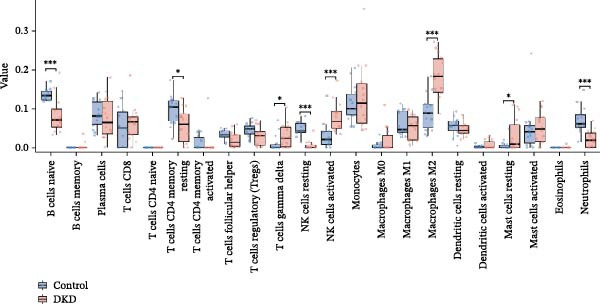
(A)

**Figure:**
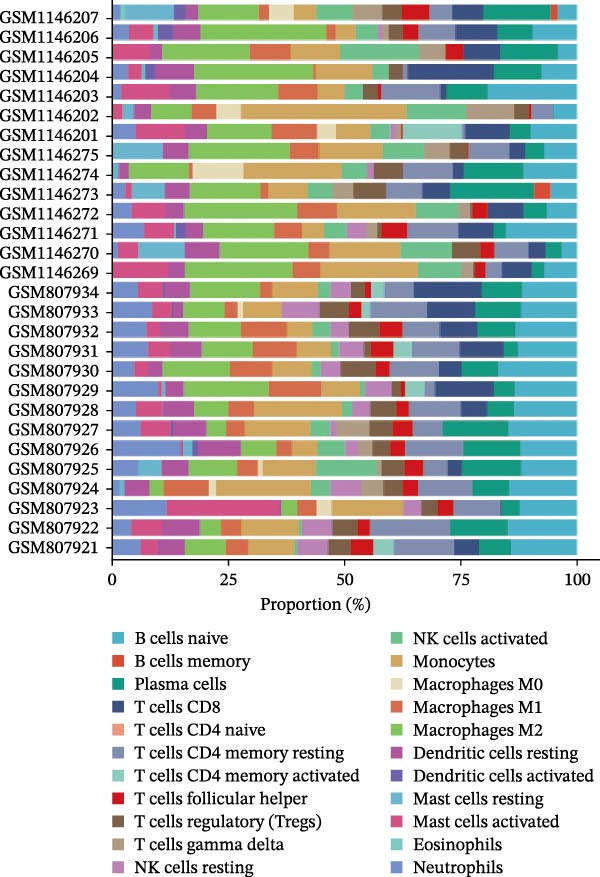
(B)

**Figure:**
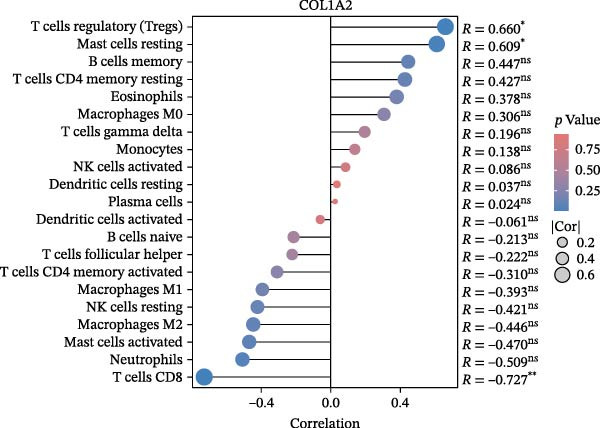
(C)

**Figure:**
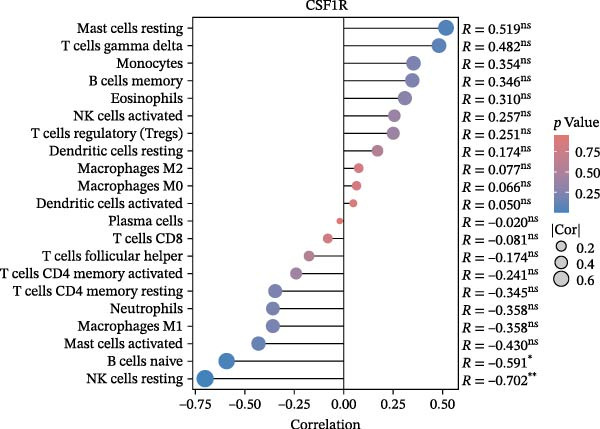
(D)

**Figure:**
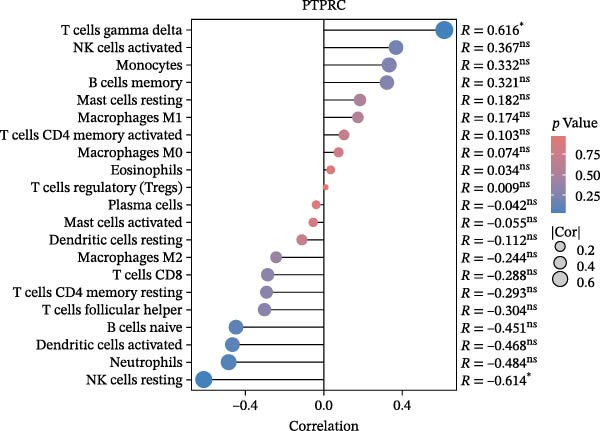
(E)

**Figure:**
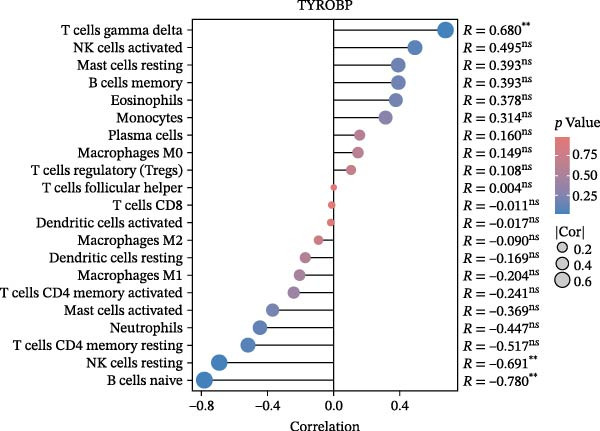
(F)

### 3.7. Single‐Nucleus Clustering Annotation and Differential Analysis

We conducted a comprehensive scRNA‐seq analysis using publicly available scRNA‐seq data from healthy and DKD kidney samples. Data on doublet detection rates and post‐filtering cell numbers per sample are provided in Supporting Information 7: File S5. The single‐cell data were processed using the standard Seurat V5 workflow. The efficacy of batch effect removal was evaluated with kBET, showing a reduction in the average rejection rate from 0.733 before integration to 0.328 after integration (Supporting Information 8: Figure S3). Subsequent clustering and annotation were then performed. Ultimately, we identified 12 major cell populations, including CNT (marked with SLC8A1), DCT (marked with SLC12A3), ENDO (marked with PTPRB and EGFL7), ICA (marked with SLC26A7), ICB (marked with SLC26A4), MES (marked with PTPRO), PC (marked with AQP2), PCT (marked with SLC5A12, SLC22A8, and SLC17A3), PEC (marked with CFH), PODO (marked with KLF6), PT (marked with MIOX and GPX3), and TAL (marked with SLC12A1). The aforementioned marker genes dot plot is shown in Supporting Information 9: Figure S4. Subsequently, we employed UMAP (Uniform Manifold Approximation and Projection) for visualization (Figure 9A,B). The bubble plot displays the marker genes used for annotation (Figure 9C). Using the FindMarkers function with thresholds of |avg_log2FC|>0.5 and *p*_val <0.05, we identified differential genes that met the criteria and presented them in a multi‐group volcano plot (Figure 9D). Notably, some of the potential marker genes for diabetic nephropathy obtained from our bulk‐RNA analysis also exhibited significant differences in specific cell populations in the single‐cell differential analysis: COL1A2 was upregulated in ENDO and PODO, while PTPRC was upregulated in PC but downregulated in ICA.

Figure 9Single‐cell cluster annotation and differential analysis. (A) Annotation of different cell types in the UMAP plot. (B) Clustering results of different cell groups at a resolution of 0.8. (C) Average expression of marker genes across different cell clusters and the proportion of cells belonging to each cluster. (D) Multi‐group differential dot plot showing differentially expressed genes across different cell types, highlighting genes such as COL1A2 and PTPRC.

**Figure:**
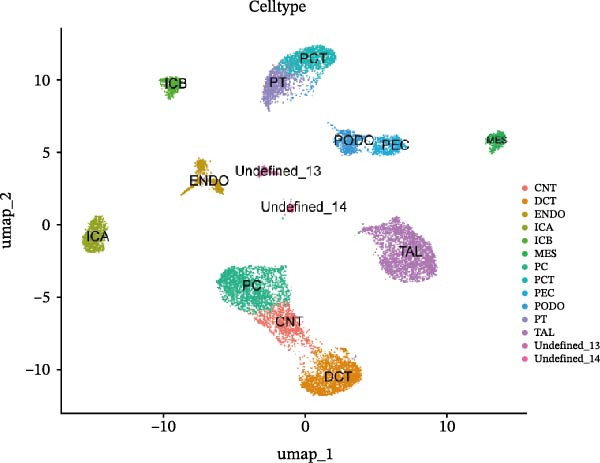
(A)

**Figure:**
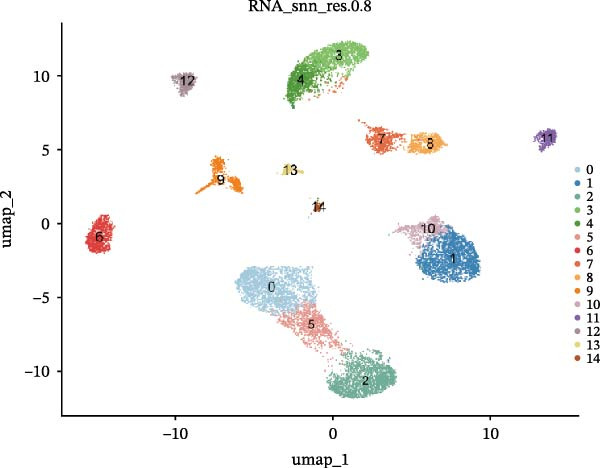
(B)

**Figure:**
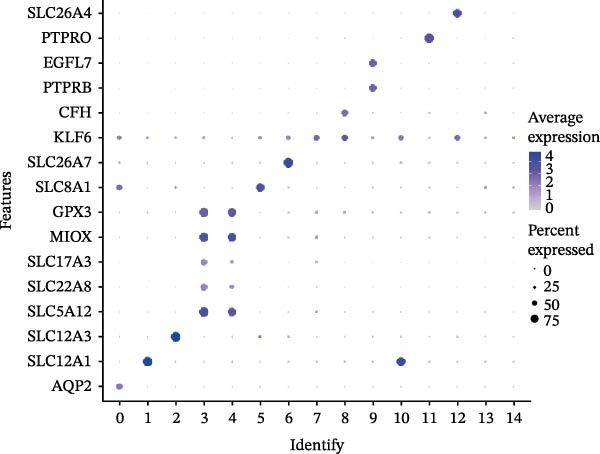
(C)

**Figure:**
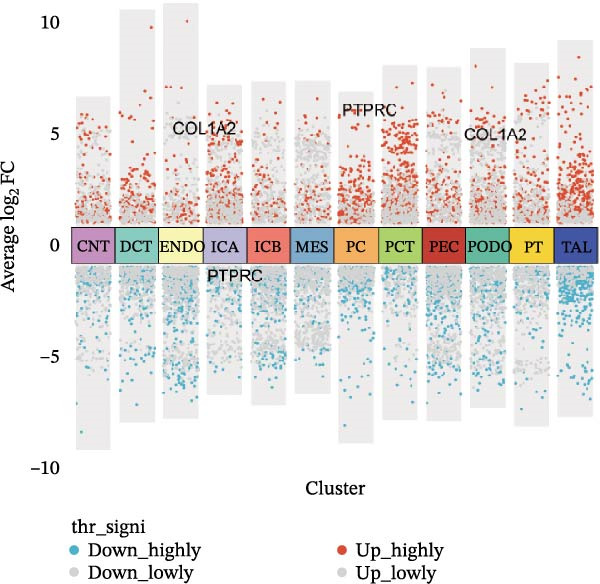
(D)

### 3.8. Cell–Cell Communication

Cell communication networks between Control and DKD cell populations were analyzed via CellChat. Circle plots visualized interaction number/intensity (Figure 10A,B). With PCT as the primary focus, the netVisual_bubble function was used to further visualize the communication between PCT and other cells, both as a signal sender (Figure 10C,E) and a signal receiver (Figure 10D,F), along with the involved ligand–receptor pairs. We visualized the SPP1, PARs, and NRG pathways using hierarchical plots, revealing significant differences between the Control and DKD groups in these signaling pathways. In the SPP1 signaling pathway, PCT in the Control group did not communicate with any other cells via the SPP1 pathway (Figure 11A), whereas in the DKD group, PCT communicated with various cells such as CNT, DCT, MES, PC, PEC, and PODO (Figure 12A). In the PARs signaling pathway, compared to the Control group, the DKD group lacked communication with PEC, but within the DKD group, PCT communicated with itself through the PARs pathway (Figures 11B, 12B). In the NRG signaling pathway, only the Control group’s PCT communicated with other cells through this pathway, while the DKD group’s PCT completely lacked communication through this pathway (Figure 11C). Subsequently, ligand–receptor pairs with differential communication between Control and DKD groups were analyzed. Differences were observed mainly in receptor pairs such as SPP1‐(ITGAV + ITGB3), SPP1‐(ITGAV + ITGB1), SPP1‐(ITGAV + ITGB5), SPP1‐(ITGAV + ITGB6), NRG1‐ERBB3, NRG1‐(ERBB2+ERBB3), NRG1‐ERBB4, NRG1‐(ERBB2+ERBB4), NRG1‐(ITGAV + ITGB3), and PLG‐PARD3. The receptor–ligand pairs that had cell communication in both groups were SPP1‐(ITGAV + ITGB3) and PLG‐PARD3, but the participating cells were different. In the Control group, the cells involved in SPP1‐(ITGAV + ITGB3) communication were DCT and PEC, while PCT did not directly communicate with other cells or itself through SPP1‐(ITGAV + ITGB3) (Figure 11D). However, in the DKD group, SPP1‐(ITGAV + ITGB3) was involved in the communication between PCT and cells such as MES, PEC, and PODO (Figure 12C). PLG‐PARD3 mediated PCT–MES–PEC–PODO communication in Control (Figure 11J); in DKD, interactions were restricted to MES–PODO (Figure 12G). The receptor–ligand pairs that only participated in PCT cell communication in the disease group were SPP1‐(ITGAV + ITGB1), SPP1‐(ITGAV + ITGB5), and SPP1‐(ITGAV + ITGB6) (Figure 12D–F), while those that only participated in PCT cell communication in the Control group were NRG1‐ERBB3, NRG1‐(ERBB2+ERBB3), NRG1‐ERBB4, NRG1‐(ERBB2+ERBB4), and NRG1‐(ITGAV + ITGB3) (Figure 11E–I). Subsequently, we visualized the contribution rankings of receptor–ligand pairs involved in the SPP1 (Supporting Information 10: Figure S5A,D), PARs (Supporting Information 10: Figure S5B, E), and NRG (Supporting Information 10: Figure S5C) pathways in the Control and DKD groups.

Figure 10CellChat cell communication analysis. (A) In the Control group, the number and strength of communications between cells (from left to right, the left plot shows the number of communications, the right plot shows the communication strength). (B) In the DKD group, the number and strength of communications between cells (from left to right, the left plot shows the number of communications, the right plot shows the communication strength). (C) In the Control group, ligand–receptor pairs involved when PCT acts as a signal sender. (D) In the Control group, ligand–receptor pairs involved when PCT acts as a signal receiver. (E) In the DKD group, ligand–receptor pairs involved when PCT acts as a signal sender. (F) In the DKD group, ligand–receptor pairs involved when PCT acts as a signal receiver.

**Figure:**
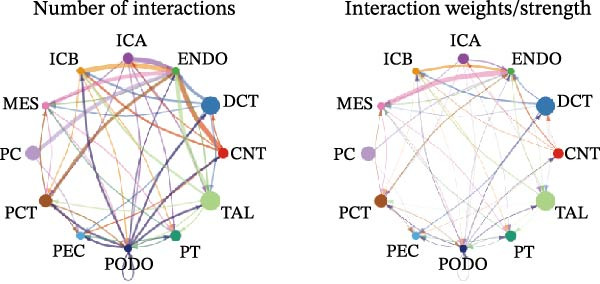
(A)

**Figure:**
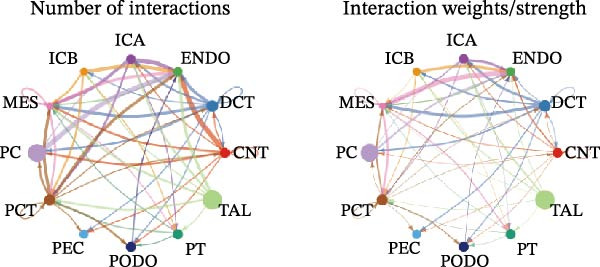
(B)

**Figure:**
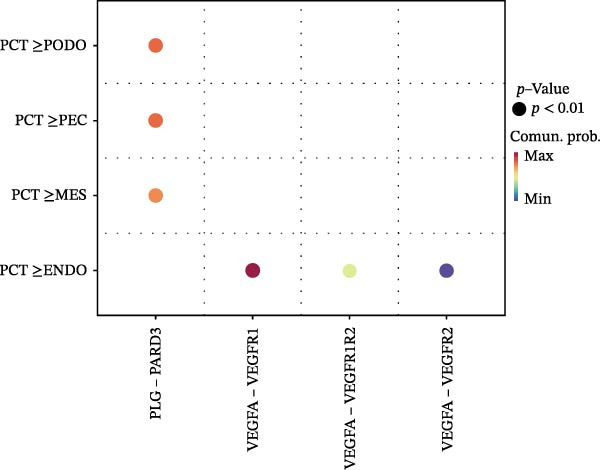
(C)

**Figure:**
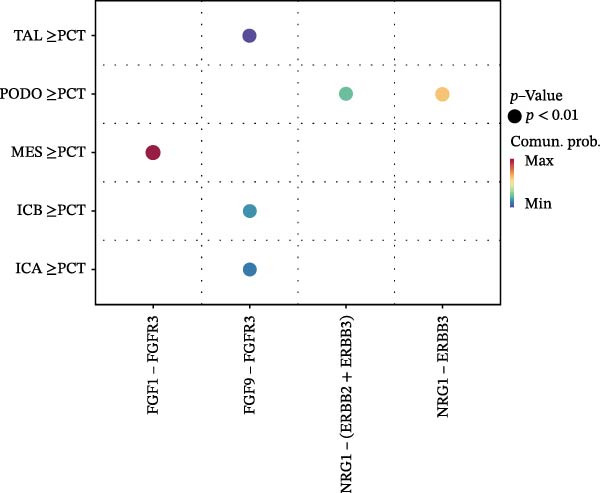
(D)

**Figure:**
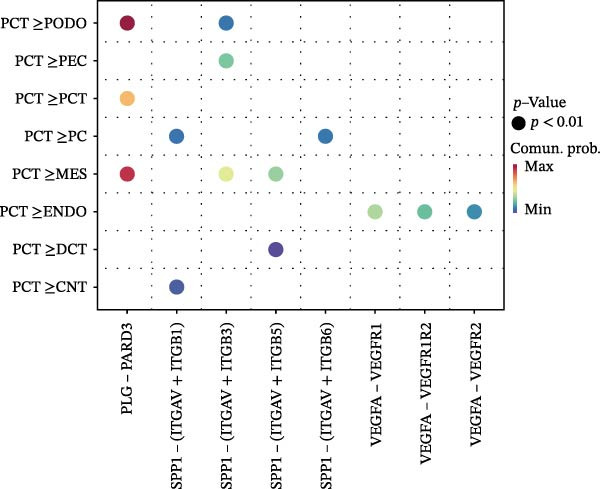
(E)

**Figure:**
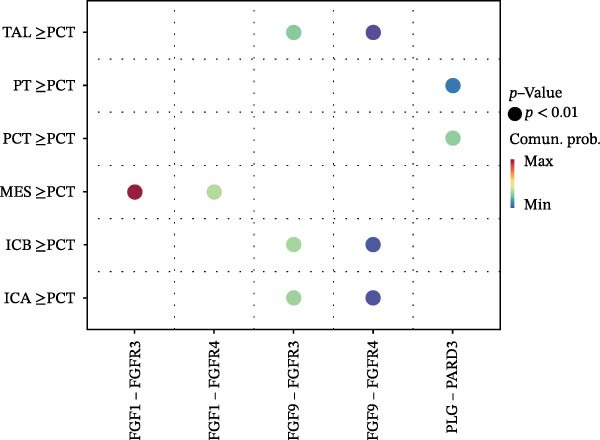
(F)

Figure 11Hierarchical plot of cell communication in the Control Group. (A) Cell types using the “SPP1 signaling pathway” for intercellular communication and their communication identities. (B) Cell types using the “PARs signaling pathway” for intercellular communication and their communication identities. (C) Cell types using the “NRG signaling pathway” for intercellular communication and their communication identities. (D) Hierarchical plot of SPP1‐related receptor–ligand pairs. (E–I) Hierarchical plots of NRG‐related receptor–ligand pairs. (J) Hierarchical plot of PARs‐related receptor–ligand pairs.

**Figure:**
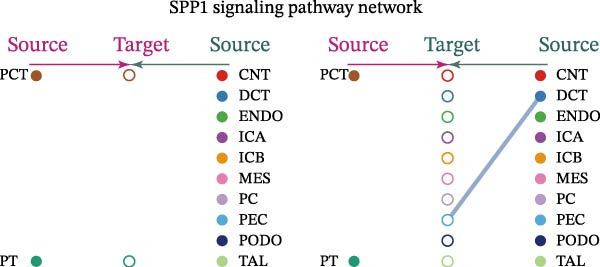
(A)

**Figure:**
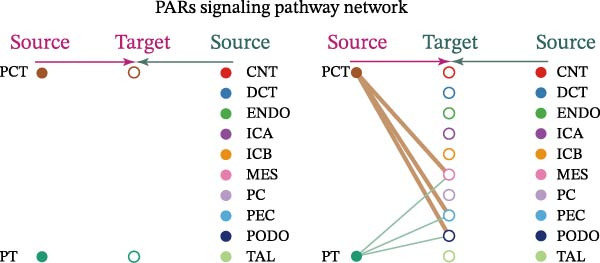
(B)

**Figure:**
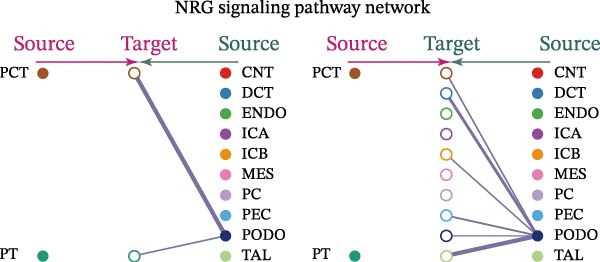
(C)

**Figure:**
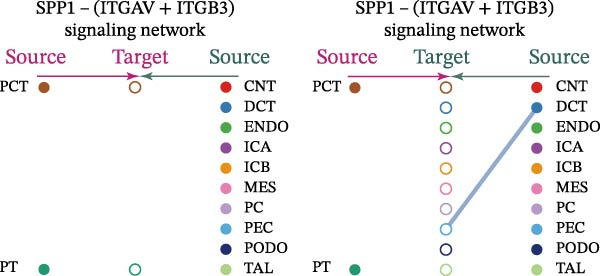
(D)

**Figure:**
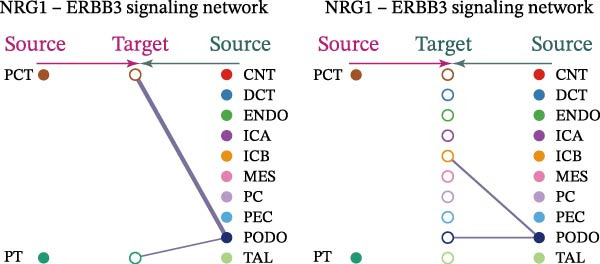
(E)

**Figure:**
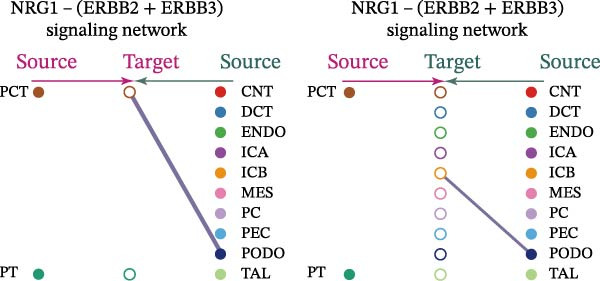
(F)

**Figure:**
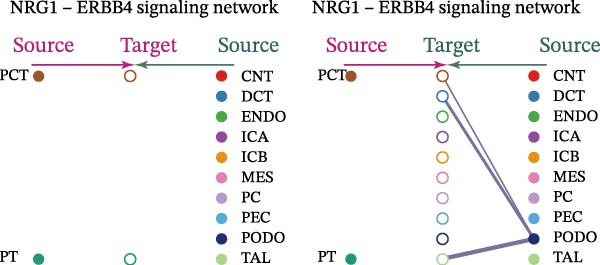
(G)

**Figure:**
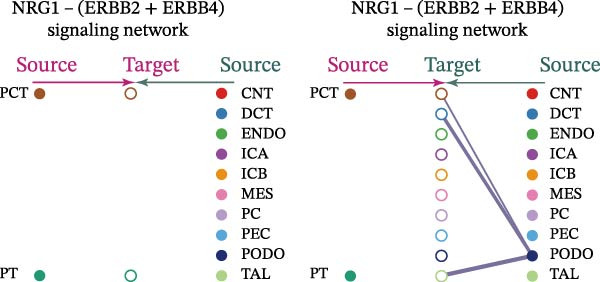
(H)

**Figure:**
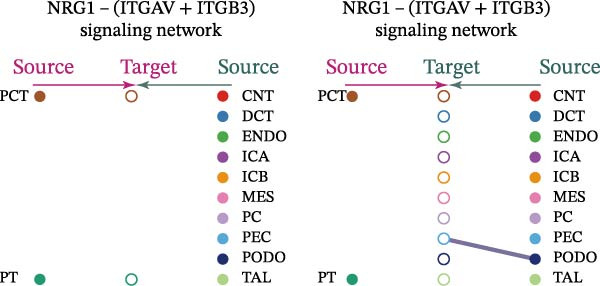
(I)

**Figure:**
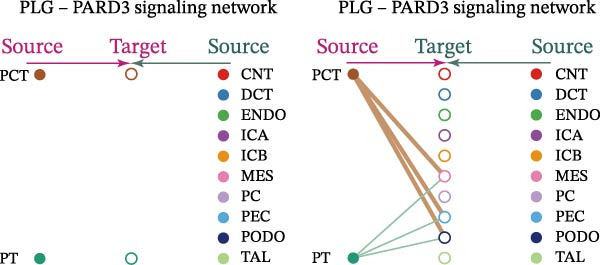
(J)

Figure 12Hierarchical plot of cell communication in the DKD group. (A) Cell types using the “SPP1 signaling pathway” for intercellular communication and their communication identities. (B) Cell types using the “PARs signaling pathway” for intercellular communication and their communication identities. (C, D, E, F) Hierarchical plots of SPP1‐related receptor–ligand pairs. (G) Hierarchical plot of PARs‐related receptor–ligand pairs.

**Figure:**
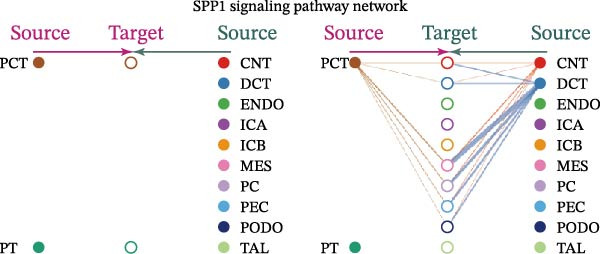
(A)

**Figure:**
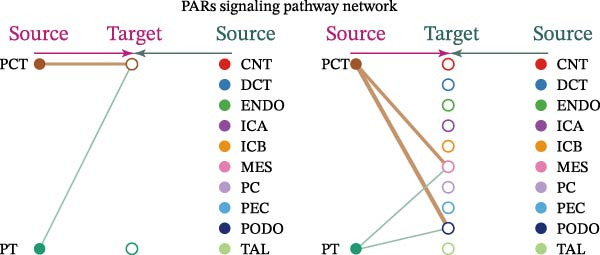
(B)

**Figure:**
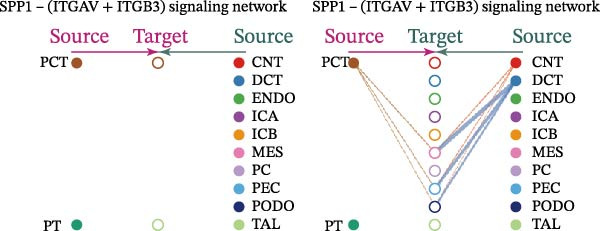
(C)

**Figure:**
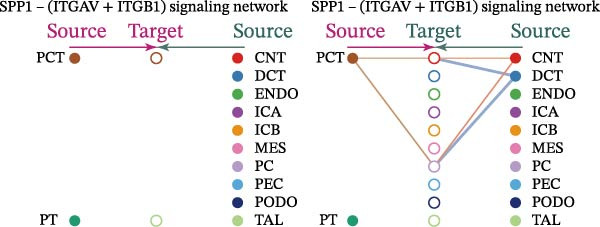
(D)

**Figure:**
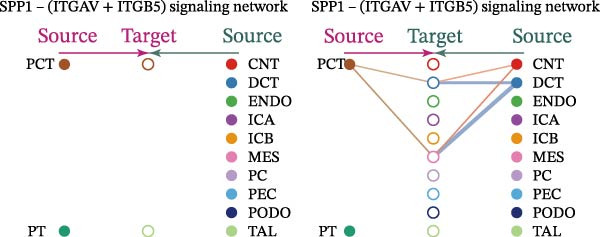
(E)

**Figure:**
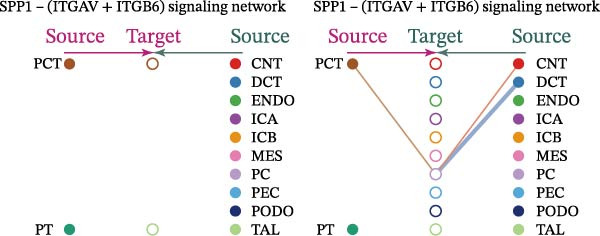
(F)

**Figure:**
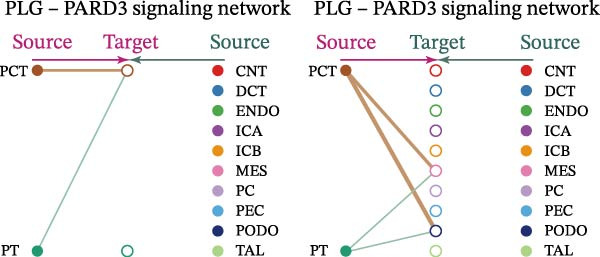
(G)

Furthermore, we analyzed cell communication patterns using NMF. PCT‐like communication cells in both Outgoing/Incoming modes differed significantly between Control and DKD groups. In Outgoing mode, the cells with the same communication pattern as PCT in the Control group were ICB, MES, and PT (Figure 13A), while in the DKD group, they were DCT, ENDO, PT, and TAL (Figure 13E). In Incoming mode, the cells with the same communication pattern as PCT in the Control group were CNT, DCT, ICB, PT, and TAL (Figure 13B), while in the DKD group, it was only PT (Figure 13F). Additionally, the contribution of signaling pathways related to PCT communication varied among different groups and communication modes. In the Control group in outgoing mode, the pathway with the greatest contribution to PCT communication was PARs (Figure 13C), while in the DKD group in Outgoing mode, it was SPP1 (Figure 13G). In the Control group in Incoming mode, the pathway with the greatest contribution was NRG (Figure 13D), whereas in the DKD group in Incoming mode, it was FGF (Figure 13H). Thus, intergroup differences in cell communication patterns and pathway contributions may drive DKD progression.

Figure 13Analysis of communication patterns. (A, B) “River plot” visualization of cell communication patterns in the “outgoing signaling” and “incoming signaling” scenarios in the Control group, depicting cell types and signaling pathways sharing similar communication patterns. (E, F) “River plot” visualization of cell communication patterns in the “outgoing signaling” and “incoming signaling” scenarios in the DKD group, depicting cell types and signaling pathways sharing similar communication patterns. (C, D) Dot plots showing the contribution of different cellular pathways in the cell communication process in the Control group. (G, H) Dot plots showing the contribution of different cellular pathways in the cell communication process in the DKD group.

**Figure:**
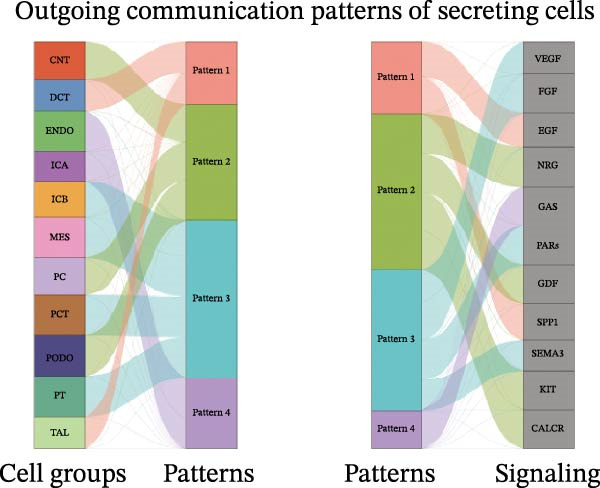
(A)

**Figure:**
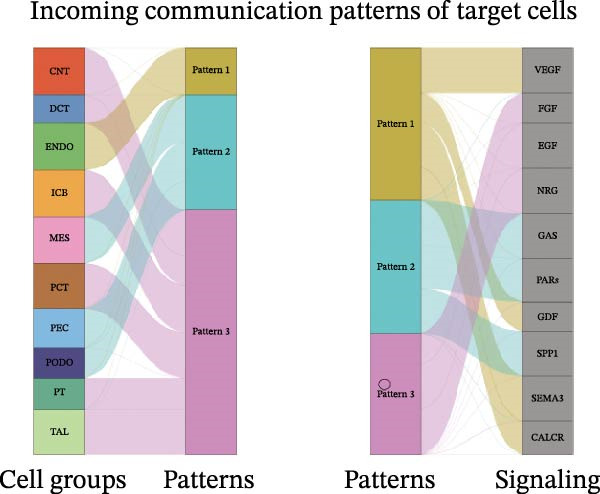
(B)

**Figure:**
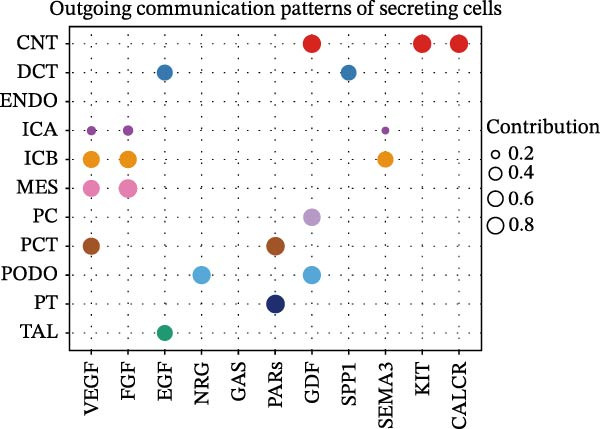
(C)

**Figure:**
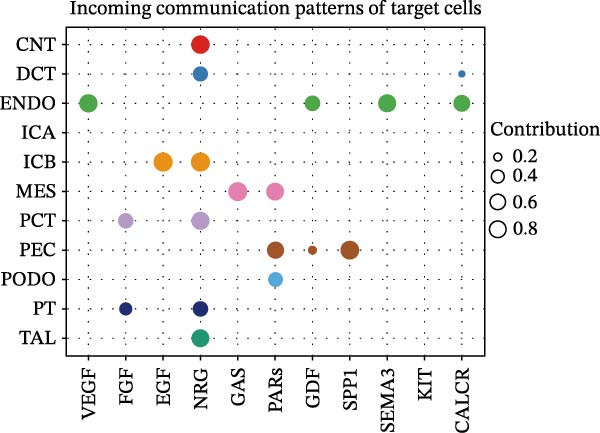
(D)

**Figure:**
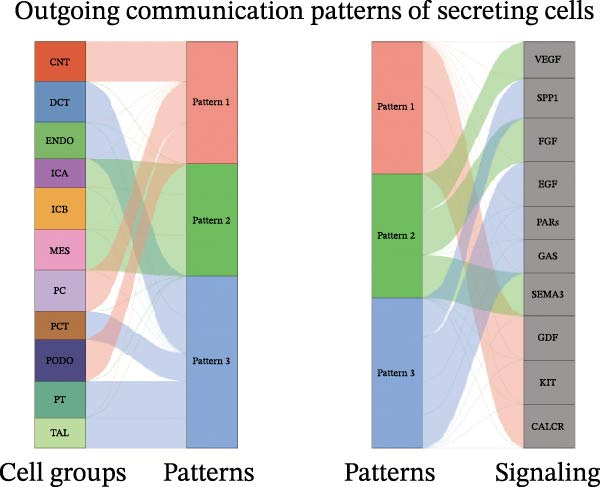
(E)

**Figure:**
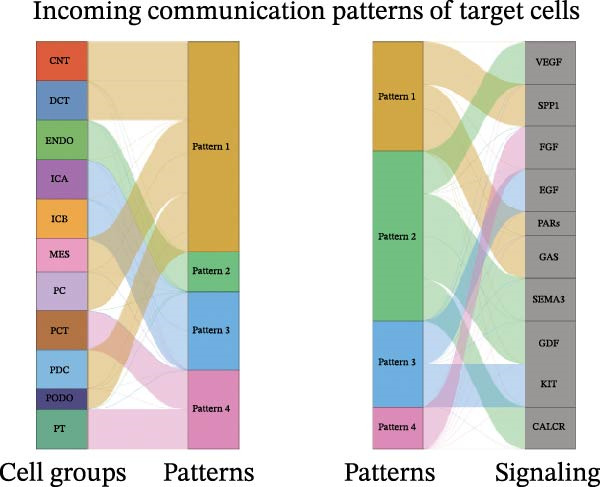
(F)

**Figure:**
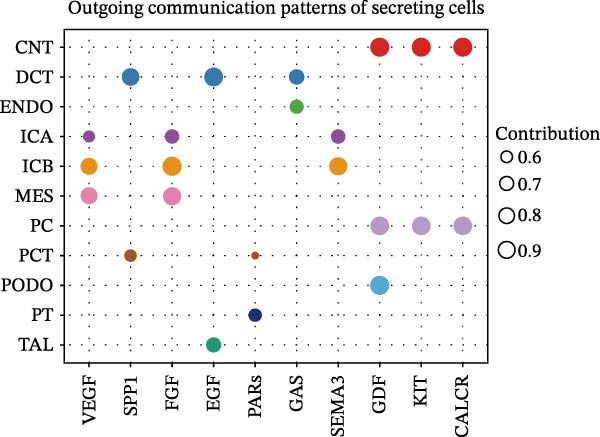
(G)

**Figure:**
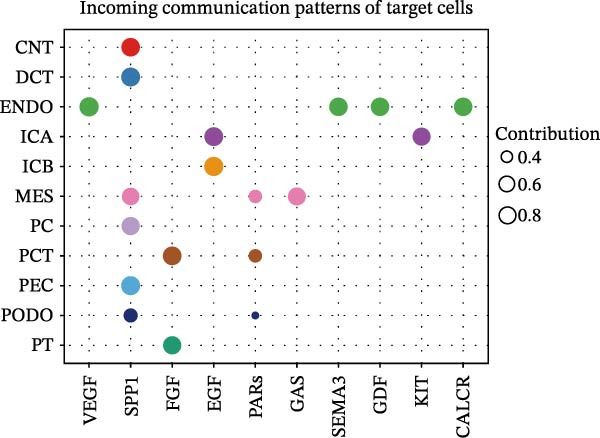
(H)

Finally, we conducted network centrality analysis on cell communication in the Control and DKD groups. We found that in the Control group, PCT served more as a signal sender than as a signal receiver (Figure 14A,C). However, in the DKD group, PCT’s roles as a signal sender and receiver were almost equal (Figure 14B,D). Additionally, as shown in Figure 14A, we observed that in the control group, the FGF and NRG pathways played dominant roles in the incoming signaling patterns, whereas in the DKD group, the dominant pathways shifted to FGF and PARs. The outgoing signaling patterns remained largely unchanged between the control and DKD groups. This suggests that the transition of PCT cells from a signal‐sending‐dominant state to a more balanced sender‐receiver state is associated with the NRG and PARs signaling pathways. Furthermore, our earlier analysis indicated that in PCT cells, the pathway contributing most significantly to the incoming pattern shifted from NRG in the control group to FGF in the DKD group (Figure 13D,F), further supporting the view that alterations in the NRG and PARs signaling pathways within the incoming signaling patterns of PCT cells are a primary cause of their changed communication mode under disease conditions.

Figure 14Network centrality analysis. (A) In the Control group, the heatmap shows the contribution of different signaling pathways to each cell type in both outgoing signaling patterns and incoming signaling patterns. The darker the color in the heatmap, the greater the contribution, indicating that this pathway plays a larger role in communication. (C) The dot plot shows the proportion of different cell types in the two communication patterns in the Control group. (B) In the DKD group, the heatmap shows the contribution of different signaling pathways to each cell type in both outgoing signaling patterns and incoming signaling patterns. The darker the color in the heatmap, the greater the contribution, indicating that this pathway plays a larger role in communication. (D) The dot plot shows the proportion of different cell types in the two communication patterns in the DKD group.

**Figure:**
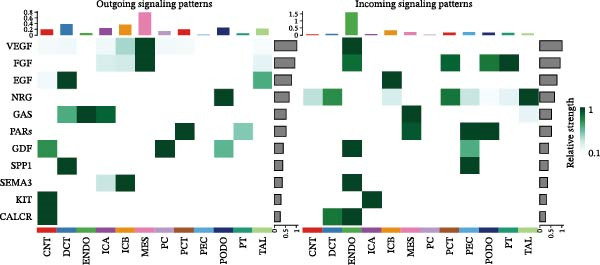
(A)

**Figure:**
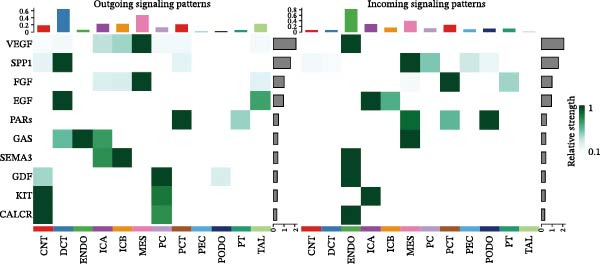
(B)

**Figure:**
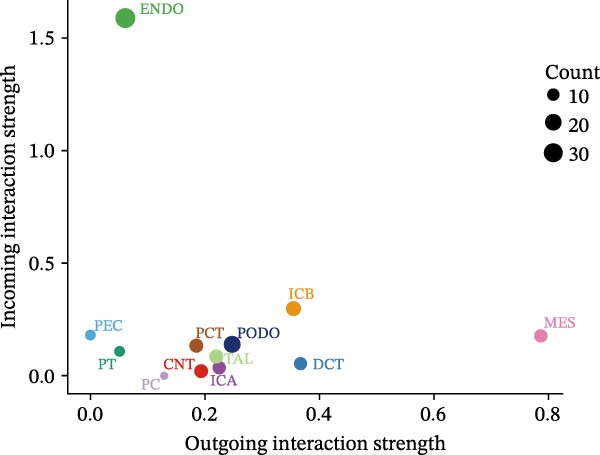
(C)

**Figure:**
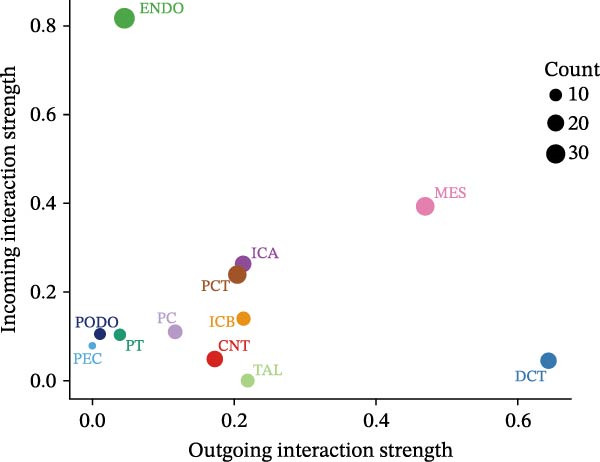
(D)

### 3.9. Pseudo‐Time Trajectory Analysis and BEAM Analysis of PCT in the Progression of DKD

We conducted a pseudo‐time trajectory analysis on PCT, dividing the entire trajectory into seven stages and three critical branch points (Figure 15A). This analysis demonstrated the distribution of PCT in the trajectory across different groups (Figure 15B) and the direction of differentiation and development (Figure 15C,D). Significant differences in the pseudo‐time trajectory distribution of PCT were observed between the Control group and the DKD group. Combining this with the direction of differentiation and development of PCT in the trajectory, it is evident that the distribution of cells in the Control group and DKD group after Branch 1 is uneven along the trajectory (Figure 15B). Pseudotime analysis of PCT revealed significantly DEGs clustered into two groups (Figure 15E). GO–KEGG enrichment identified cluster‐specific terms: BP (P.adj‐filtered)—Import across plasma membrane, anion/carboxylic acid transmembrane transport; MF—secondary active/solute: cation/sodium: phosphate symporter activity; CC—apical/basal plasma membrane (Figure 15F).

Figure 15Pseudotime trajectory analysis. (A) Seven stages and three critical branch points of PCT in pseudotime trajectory analysis. (B) Distribution of PCT along the trajectory under different groupings. (C) Differentiation and development directions of PCT in the pseudotime trajectory. (D) Cell density curves of PCT across different groups along pseudotime trajectories. (E) Two expression patterns of significantly DEGs. (F) GO/KEGG enrichment analysis of significantly DEGs in different patterns. (G) Three expression patterns of potential regulatory genes at the branching points. (H) GO/KEGG enrichment analysis of significantly DEGs in different.

**Figure:**
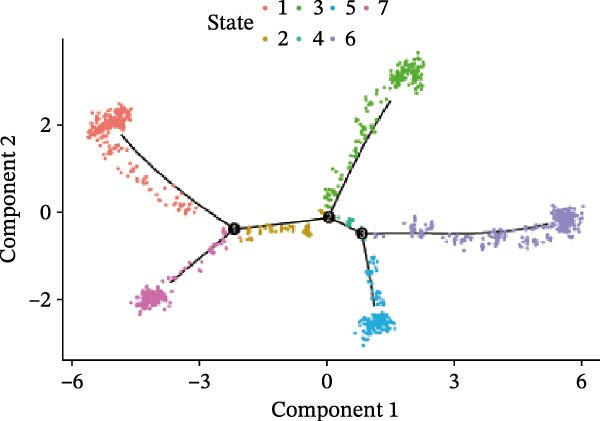
(A)

**Figure:**
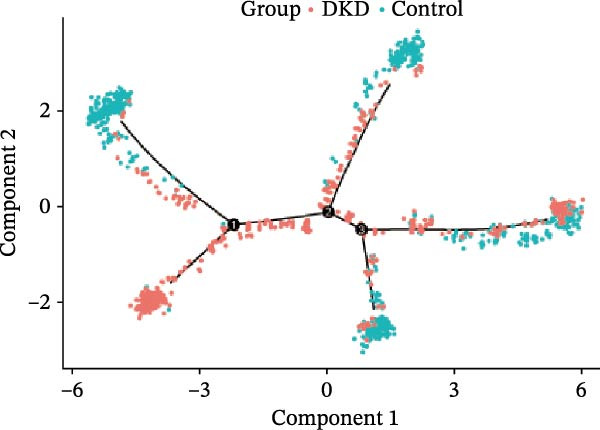
(B)

**Figure:**
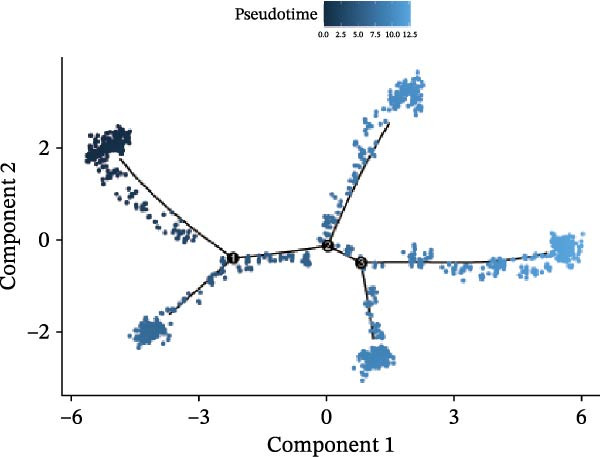
(C)

**Figure:**
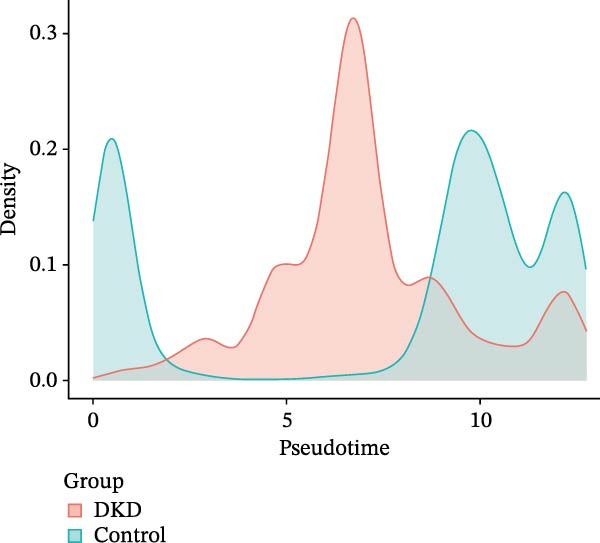
(D)

**Figure:**
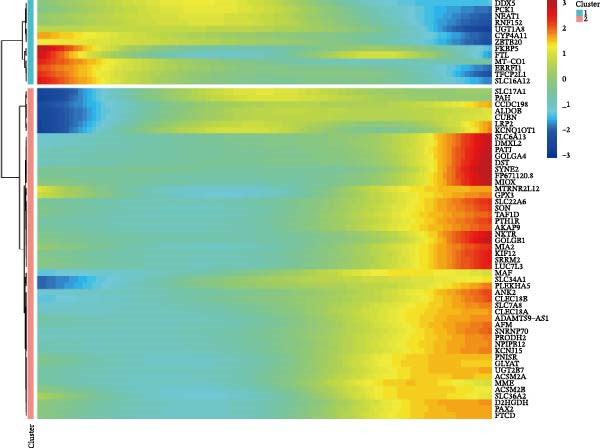
(E)

**Figure:**
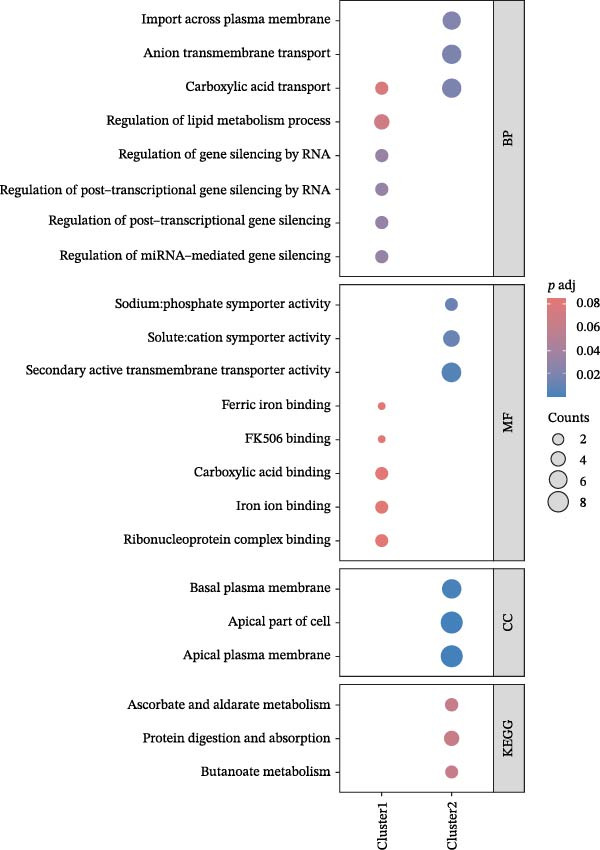
(F)

**Figure:**
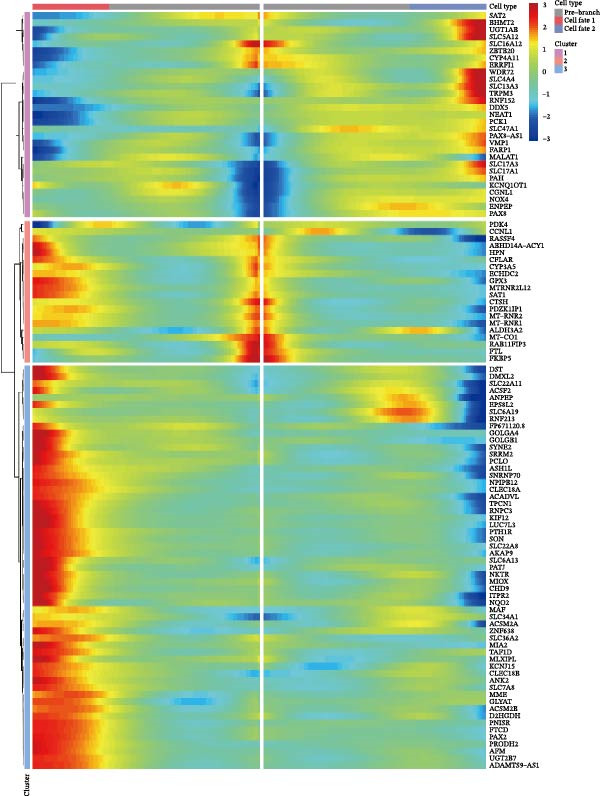
(G)

**Figure:**
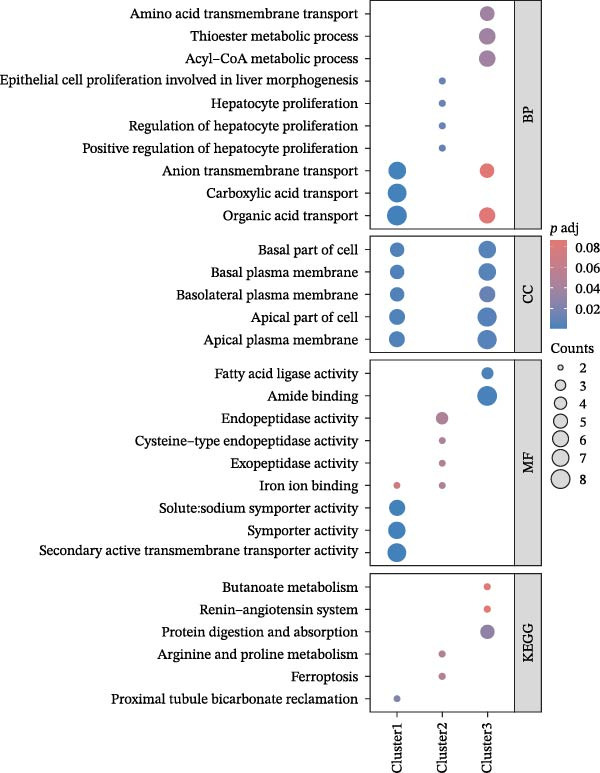
(H)

Subsequently, through BEAM analysis of Branch 1, we identified potential regulatory genes that govern cell differentiation and development at Branch 1 during the pseudo‐time process of PCT. Based on different expression patterns, these genes were clustered into three distinct clusters (Figure 15G). GO and KEGG enrichment analyses were conducted again to identify potential pathways and related biological functions that regulate the pseudo‐time trajectory of cells at Branch 1 under the three different patterns. By screening the results using P.adj, we obtained BP such as organic anion transport; MF such as secondary active transmembrane transporter activity; CC such as apical plasma membrane; and pathways such as Proximal tubule bicarbonate reclamation, ferroptosis, arginine and proline metabolism, and protein digestion and absorption (Figure 15H).

## 4. Discussion

This study has made significant progress in exploring the pathogenesis of DKD. By integrating transcriptome data with single‐cell data analysis, we have not only identified COL1A2, CSF1R, PTPRC, and TYROBP as potential diagnostic biomarkers for DKD but also profoundly unveiled the pivotal role of PCT cells in the progression of DKD. Firstly, regarding the research on COL1A2, CSF1R, PTPRC, and TYROBP as potential diagnostic biomarkers, these genes exhibit significant differential expression during the pathogenesis of DKD. Through enrichment analysis, PPI network construction, and other methods, we have confirmed their close association with the pathogenesis of DKD. The discovery of these biomarkers not only provides new clues for the early diagnosis of DKD but also offers powerful support for disease monitoring and prognosis assessment. Clinically, these biomarkers have the potential to become important indicators for assessing the progression of DKD in patients and guiding personalized treatment. Previous study employed bioinformatics methods to identify COL1A as a possible crucial gene linked to the advancement of DKD [55]. Further research using WGCNA found that the association between COL1A2 and DKD may be related to inflammation and fibrosis [56]. Previous studies on CSF1R have shown that its expression is significantly upregulated in DKD and may serve as a potential biomarker for the condition [57]. Landscape studies on infiltrating immune cells and related genes in diabetic nephropathy have identified TYROBP as a key immune regulatory gene [58]. Thus, consistent with the conclusions of this study, previous literature has reported close associations between COL1A2, CSF1R, PTPRC, and TYROBP with DKD. However, the mechanisms reported mainly focus on the regulation of immune inflammation and fibrosis. What distinguishes this study from others is that it has, to our knowledge, discovered that COL1A2, CSF1R, PTPRC, and TYROBP may influence the progression of DKD by affecting autophagy, providing new directions and insights into the progression and treatment of DKD.

PCT cells, as crucial cell types in the kidney, are crucial for preserving kidney structure and function [59]. This study, through pseudo‐time trajectory analysis, has revealed previously uncharacterized dynamic change patterns of PCT cells during the progression of DKD. We found that as DKD develops, PCT cells demonstrate obvious heterogeneity, with significant changes in their number, morphology, and function. This insight deepens the knowledge of PCT cells in DKD and introduces a novel angle for understanding DKD’s pathogenesis. In the future, in‐depth research on the specific mechanisms of action of PCT cells in DKD is expected to provide new therapeutic targets and strategies for the treatment of DKD. Other studies have reported that increased expression of KIM‐1 in PCT cells mediates the absorption of albumin bound to palmitic acid by PCT cells, leading to more severe tubular injury, DNA damage, PCT cell cycle pause, interstitial inflammation, and fibrosis, as well as secondary glomerulosclerosis, ultimately facilitating the advancement of DKD [60]. Additional research has found that activation of the p53/miR‐214/ULK1 axis in PCTs results in autophagy dysregulation, exacerbating PCT injury, inflammation, and fibrosis, thereby facilitating the progression of DKD [61]. Recent studies have discovered that ER phagocytosis in tubular cells of STZ‐induced diabetic mice is severely impaired, with decreased expression of PACS‐2, inhibiting autophagy, and exacerbating tubular injury in DKD [62]. Thus, it can be seen that PCT cells are essential in the onset and development of DKD, and autophagy is likely a novel biological mechanism underlying the development of PCTs and DKD.

Single‐cell data analysis revealed that in DKD, PCT transition from a signal‐sending‐dominant state (Control) to a balanced state of both signal sending and receiving. This suggests a shift from actively regulating the microenvironment to passively responding to disease stimuli. Such a transition may reflect alterations in cellular states caused by energy metabolism imbalance (such as mitochondrial dysfunction) in DKD [63]. Previous studies have shown that renal tubular epithelial cells undergo metabolic reprograming in chronic kidney disease, mainly manifested as mitochondrial metabolic disorders and inhibited fatty acid metabolism [64], which are associated with the progression of fibrosis and have been considered core mechanisms of tubular injury in DKD [65]. In future research, exploring the mechanisms underlying energy metabolism imbalance in PCT will contribute to unraveling the causes of fibrosis in DKD.

Further analysis combined with single‐cell data suggests that in the PCT of the DKD group, the SPP1 signaling pathway is significantly activated, and SPP1 interacts with MES via the SPP1‐(ITGAV + ITGB1/3/5/6) receptor pair, a phenomenon not observed in the control group. Based on previous research findings, SPP1 functions as a secreted cytokine that activates downstream signals (such as FAK‐YAP) [66] by binding to integrin receptors (e.g., ITGAV + ITGB1), driving the fibrotic phenotype of MES [67, 68]. In this study, COL1A2 was found to be significantly enriched in ECM–receptor interaction and focal adhesion pathways in transcriptome data, suggesting its direct involvement in ECM synthesis. COL1A2 is a core component of type I collagen, and its high expression is a hallmark of fibrosis [69]. The activation of MES (via SPP1 signaling) in single‐cell data aligns temporally and spatially with the high expression of COL1A2 in transcriptome data, hinting that MES is the primary source of COL1A2. Additionally, immune infiltration analysis revealed a positive correlation between COL1A2 expression and the infiltration of Tregs (*R* = 0.66) and M2 macrophages, as well as a negative correlation with CD8 T cells (*R* = −0.73). M2 macrophages inhibit CD8 T cell function by secreting TGF‐β/IL‐10, suggesting that COL1A2 may directly promote collagen deposition to form a physical barrier, limiting CD8 T cell infiltration, and may also lead to ECM remodeling and the release of cytokines (such as TGF‐β), inducing the differentiation of Tregs/M2 and creating an immunosuppressive microenvironment. Therefore, through integrated multi‐omics analysis, our study suggests that the SPP1 signaling pathway promotes renal fibrosis in DKD by driving the synergistic effects of ECM remodeling and immunosuppression. Based on our CellChat analysis, we observed a notable shift in the inferred signaling patterns of PCT cells between control and DKD conditions. Specifically, while the FGF and NRG pathways dominated the incoming signaling patterns in control PCT cells, the dominant incoming pathways shifted to FGF and PARs in DKD PCT cells, with the outgoing patterns remaining largely consistent. This suggests that the transition of PCT cells from a primarily signal‐sending state to a more balanced sender‐receiver state is associated with alterations in the NRG and PARs signaling pathways. The biological relevance of this shift can be contextualized by existing literature on these pathways in kidney pathophysiology. The NRG signaling axis, particularly through its ligand NRG‐1, has been demonstrated to exert protective effects in chronic kidney disease models, improving renal function and mitigating cardiac complications associated with uremic cardiomyopathy [70]. Its dominant role in the incoming signals of healthy PCT cells may reflect a tonic, protective communication necessary for maintaining tubular epithelial homeostasis and facilitating repair responses. Conversely, the emergence of PARs signaling as a major incoming pathway in DKD PCT cells aligns with established pro‐fibrotic and pro‐inflammatory roles of this receptor family, especially PAR2. Evidence indicates that PAR2 activation in renal tubules drives cellular senescence, suppresses fatty acid oxidation, and promotes the secretion of inflammatory and fibrotic factors, thereby accelerating kidney injury and fibrosis [71]. Therefore, the increased reliance on PARs signaling in DKD likely signifies a pathological rewiring of PCT cell communication. This shift may render PCT cells more receptive to microenvironmental cues that promote inflammation and matrix deposition, hallmarks of DKD progression. The transition from NRG‐dominated to PARs‐dominated incoming signaling in PCT cells may represent a switch from a protective/homeostatic communication mode to a maladaptive, disease‐promoting one. This altered signaling crosstalk could be a key mechanism underlying the compromised PCT cell function and enhanced susceptibility to fibrosis observed in DKD. Future studies specifically modulating these pathways in PCT cells are warranted to establish their causal roles in the observed cellular communication shift and disease pathogenesis.

Furthermore, the application of BEAM analysis has further deepened our understanding of the pathogenesis of DKD. Through BEAM analysis, we have unveiled the expression patterns and biological significance of DKD‐related genes within cellular populations. These findings not only aid in comprehending the gene regulatory networks during the pathogenesis of DKD but also provide clues for identifying novel therapeutic targets. However, this study still has certain limitations. For instance, we have only conducted pseudotime trajectory analysis on PCT so far and have not delved into other potential cell types or molecular markers. In the future, we can further broaden the scope of our research by conducting similar analyses on other key cell types or molecular markers to more comprehensively uncover the pathogenesis of DKD. Additionally, we can integrate more clinical data and biological experimental validations to further verify the effectiveness and safety of the potential therapeutic targets identified in this study. Through these supplementary studies, we can provide more precise and efficient strategies for treating DKD.

## Author Contributions

**Qin Wang**: validation, visualization, writing – review & editing, investigation. **Wen Ye**: formal analysis, investigation, writing – review & editing. **Xiaoqi Li**: writing – review & editing, validation, investigation. **Qi Wang**: conceptualization, formal analysis, funding acquisition, project administration. **Xianjin Bi**: resources, supervision, validation, visualization, writing – original draft.

## Funding

This work was supported by research grants from Xinjiang Uygur Autonomous Region health care research project (BL202436), the Natural Science Foundation of China (No. 82200836) and the General Program of Natural Science Foundation of Chongqing (No. CSTB2022NSCQ‐MSX1195).

## Ethics Statement

The animal ethics protocol was approved by the Medical Ethics Committee of the Second Affiliated Hospital of Xinjiang Medical University, with the ethics approval number: KY2024102828.

## Conflicts of Interest

The authors declare no conflicts of interest.

## Supporting Information

Additional supporting information can be found online in the Supporting Information section.

## Supporting information


**Supporting Information 1** File S1. Summary of all entries from GO/KEGG enrichment analysis.

**Supporting Information 2** Figure S1. Comparison of cellular features before and after quality control filtering. (A) Distribution of nFeature_RNA, nCount_RNA, and percent_mito across cells before quality control. (B) Distribution after filtering cells based on nFeature_RNA (500‐3000), nCount_RNA (<6000), and percent_mito (<5%).

**Supporting Information 3** File S2. The dimension‐reduced coordinates of Principal Component 1 (PC1) and Principal Component 2 (PC2) for each sample.

**Supporting Information 4** File S3. Complete list of DEGs.

**Supporting Information 5** File S4. ROC‐related statistics.

**Supporting Information 6** Figure S2. Scatter plots. correlations between COL1A2 (A–C), CSF1R (D–E), PTPRC (F–G), TYROBP (H–J) and immune cells. (K) Correlation network heatmap displaying the correlations among different immune cells.

**Supporting Information 7** File S5. Doublet detection rate during the doublet removal process and post‐filter cell count for each sample.

**Supporting Information 8** Figure S3. Assessment of batch effect removal by kBET analysis. (A) Before Harmony integration, the average rejection rate across all samples was 0.733, indicating pronounced batch effects. (B) After Harmony integration, the average rejection rate decreased to 0.328 across all samples, demonstrating effective batch effect removal.

**Supporting Information 9** Figure S4. Dot plots of canonical marker genes. Each dot in the plot represents a cell, with darker red indicating higher expression of the marker gene in that cell.

**Supporting Information 10** Figure S5. Contribution rankings of receptor–ligand pairs. (A, D) Contribution rankings of SPP1‐related receptor–ligand pairs in the Control and DKD groups. (B, E) Contribution rankings of PARs‐related receptor–ligand pairs in the Control and DKD groups. (C) Contribution ranking of NRG‐related receptor–ligand pairs in the Control group.

## Data Availability

The original contributions presented in the study are included in the article/Supporting Information. Further inquiries can be directed to the corresponding author.
