# Supplementary figures and images for: Screening Autophagy‐Related DKD Biomarkers Based on Bulk RNA and Single‐Cell Analysis and Uncovering Their Regulatory Mechanisms in DKD Renal Fibrosis
Source: J Diabetes Res. 2026 Apr 7;2026:3768039. doi: 10.1155/jdr/3768039 (PMC13054842; doi:10.1155/jdr/3768039)

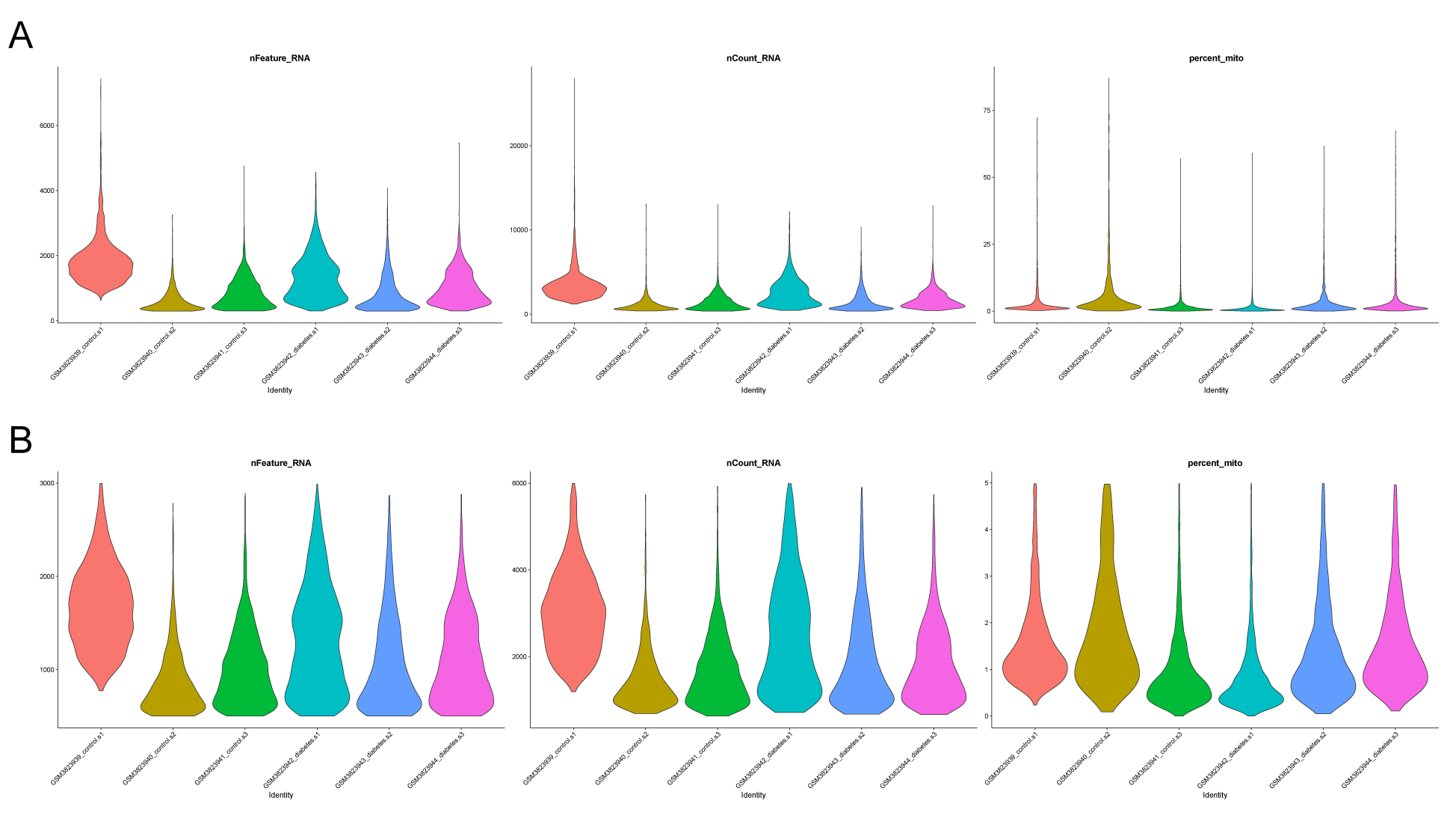

Supplement: Supplementary file 2 — Supporting Information 2 Figure S1. Comparison of cellular features before and after quality control filtering. (A) Distribution of nFeature_RNA, nCount_RNA, and percent_mito across cells before quality control. (B) Distribution after filtering cells based on nFeature_RNA (500‐3000), nCount_RNA (<6000), and percent_mito (<5%). [file JDR-2026-3768039-s002.tif]

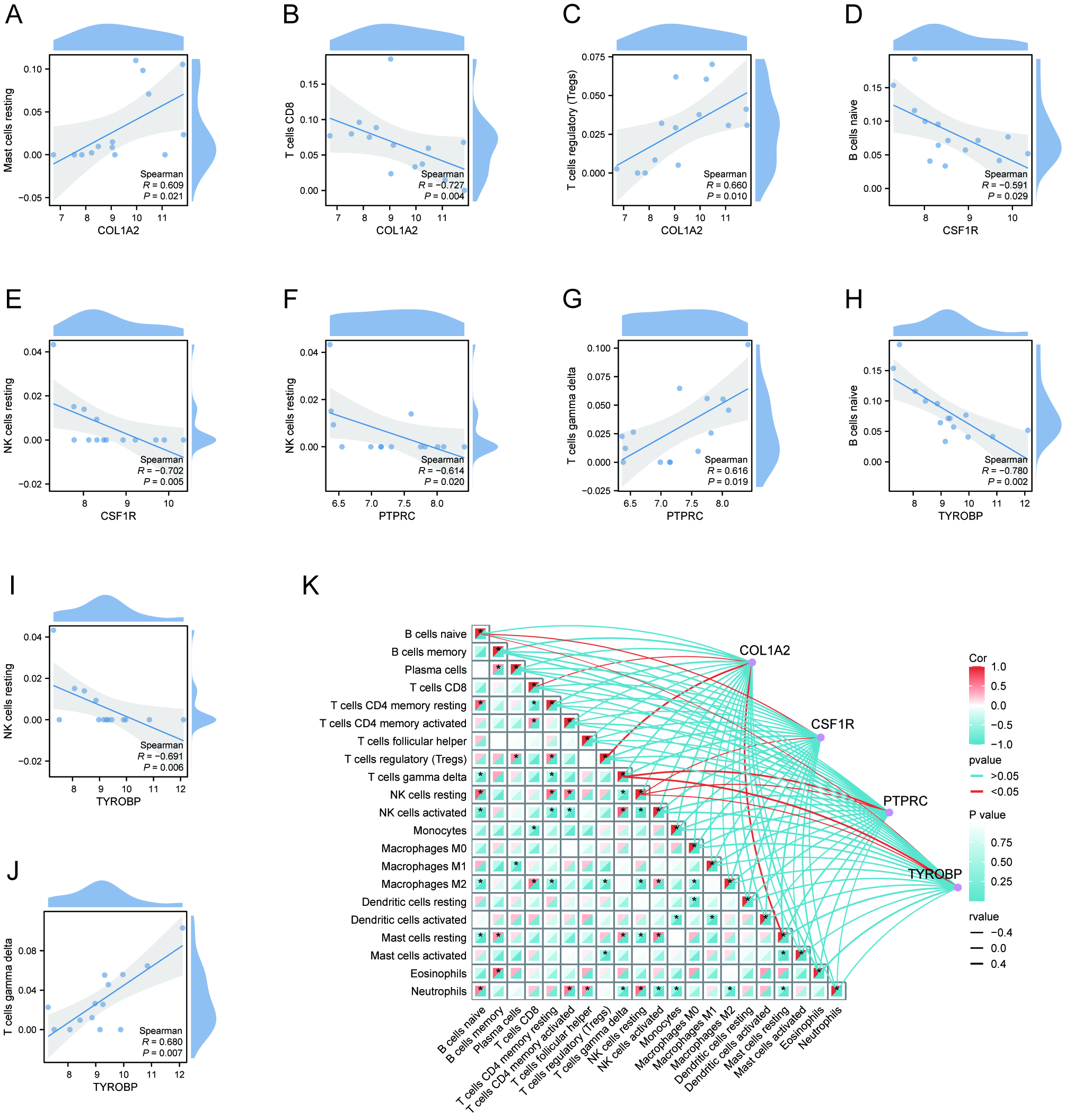

Supplement: Supplementary file 6 — Supporting Information 6 Figure S2. Scatter plots. correlations between COL1A2 (A–C), CSF1R (D–E), PTPRC (F–G), TYROBP (H–J) and immune cells. (K) Correlation network heatmap displaying the correlations among different immune cells. [file JDR-2026-3768039-s006.tif]

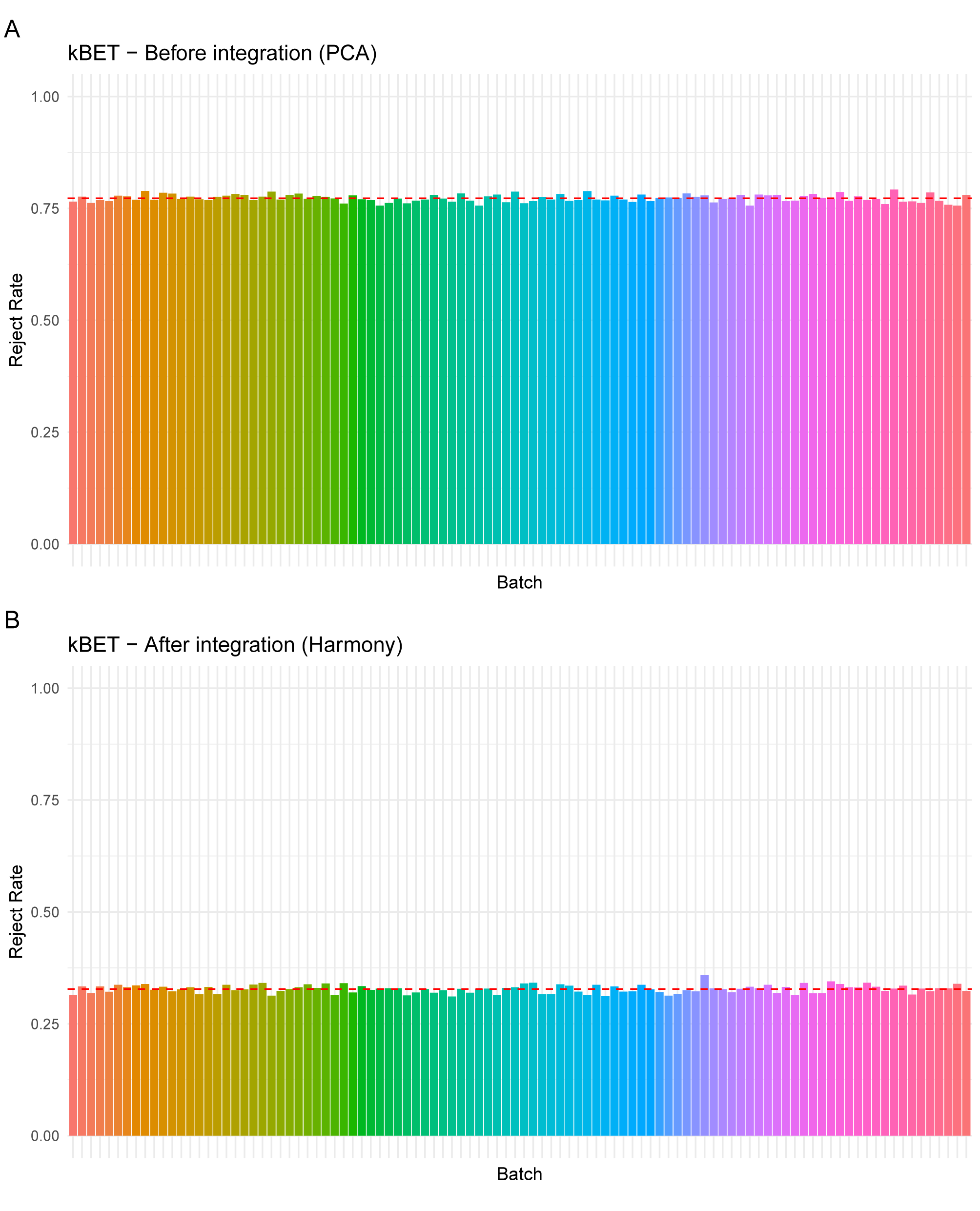

Supplement: Supplementary file 8 — Supporting Information 8 Figure S3. Assessment of batch effect removal by kBET analysis. (A) Before Harmony integration, the average rejection rate across all samples was 0.733, indicating pronounced batch effects. (B) After Harmony integration, the average rejection rate decreased to 0.328 across all samples, demonstrating effective batch effect removal. [file JDR-2026-3768039-s008.tif]

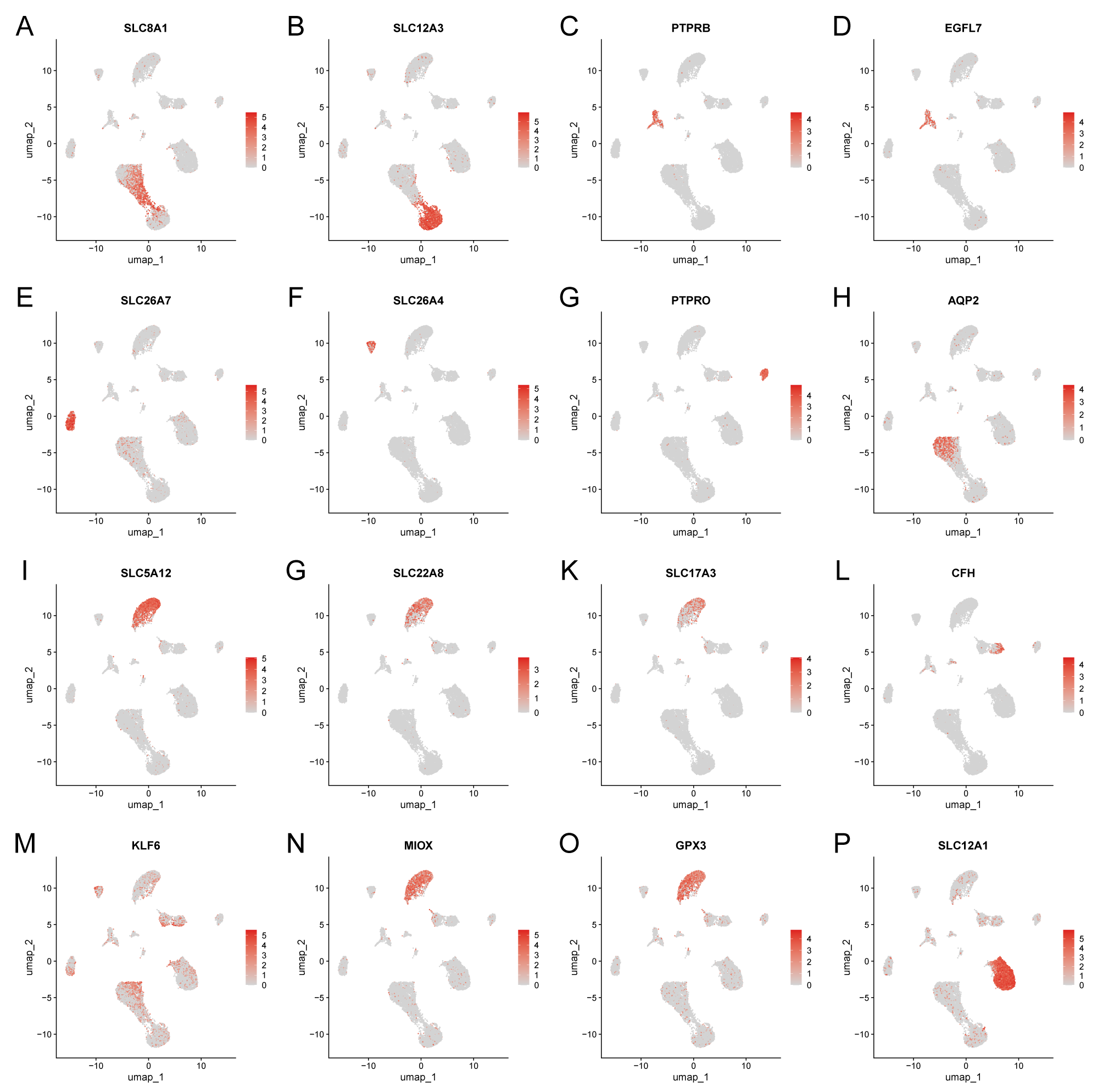

Supplement: Supplementary file 9 — Supporting Information 9 Figure S4. Dot plots of canonical marker genes. Each dot in the plot represents a cell, with darker red indicating higher expression of the marker gene in that cell. [file JDR-2026-3768039-s009.tif]

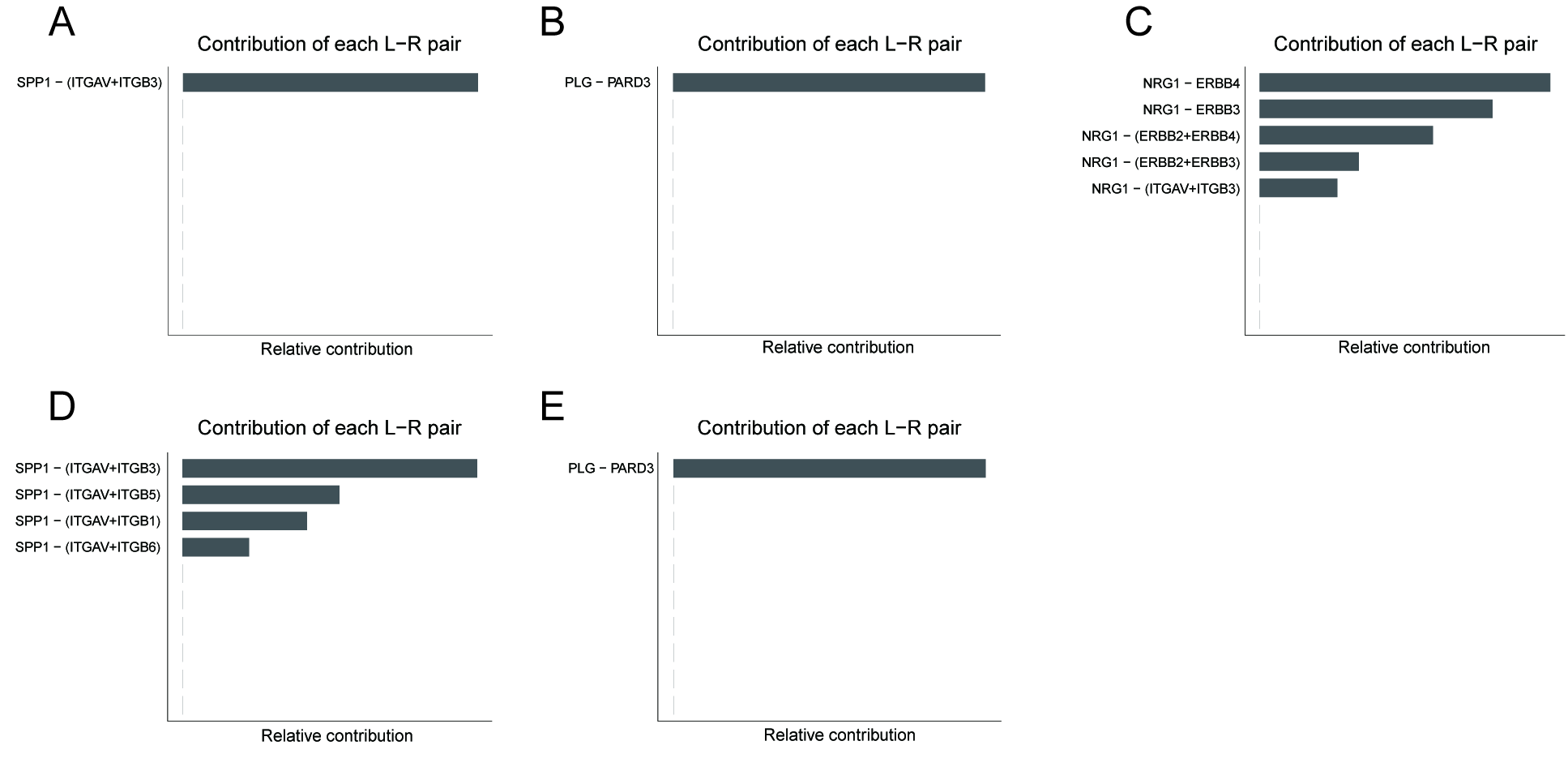

Supplement: Supplementary file 10 — Supporting Information 10 Figure S5. Contribution rankings of receptor–ligand pairs. (A, D) Contribution rankings of SPP1‐related receptor–ligand pairs in the Control and DKD groups. (B, E) Contribution rankings of PARs‐related receptor–ligand pairs in the Control and DKD groups. (C) Contribution ranking of NRG‐related receptor–ligand pairs in the Control group. [file JDR-2026-3768039-s005.tif]
